# Identifying ligands for the PHD1 finger of KDM5A through high-throughput screening†

**DOI:** 10.1039/d3cb00214d

**Published:** 2023-12-18

**Authors:** Gloria Ortiz, James E. Longbotham, Sophia L. Qin, Meng Yao Zhang, Gregory M. Lee, R. Jeffrey Neitz, Mark J. S. Kelly, Michelle R. Arkin, Danica Galonić Fujimori

**Affiliations:** a Department of Cellular and Molecular Pharmacology, University of California San Francisco San Francisco CA 94158 USA danica.fujimori@ucsf.edu; b Small Molecule Discovery Center (SMDC), University of California San Francisco San Francisco CA 94158 USA; c Department of Pharmaceutical Chemistry, University of California San Francisco San Francisco CA 94158 USA

## Abstract

PHD fingers are a type of chromatin reader that primarily recognize chromatin as a function of lysine methylation state. Dysregulated PHD fingers are implicated in various human diseases, including acute myeloid leukemia. Targeting PHD fingers with small molecules is considered challenging as their histone tail binding pockets are often shallow and surface-exposed. The KDM5A PHD1 finger regulates the catalytic activity of KDM5A, an epigenetic enzyme often misregulated in cancers. To identify ligands that disrupt the PHD1-histone peptide interaction, we conducted a high-throughput screen and validated hits by orthogonal methods. We further elucidated structure–activity relationships in two classes of compounds to identify features important for binding. Our investigation offers a starting point for further optimization of small molecule PHD1 ligands.

## Abstract 2

To identify ligands for the regulatory PHD1 domain of KDM5A, high-throughput screening and biophysical validation were conducted and led to the identification of benzofuran ligands. This work offers a starting point for further optimization of PHD1 ligands.

## Graphical abstract

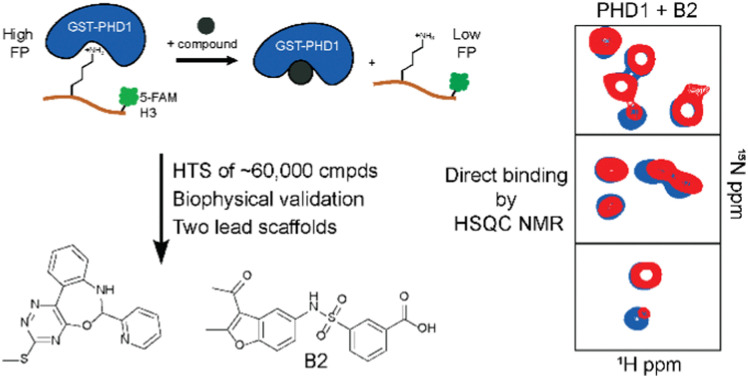

## Introduction

By recognizing specific post-translational modifications on histone proteins, chromatin reader domains recruit and/or stabilize interactions of transcription regulators with chromatin. Plant homeodomain (PHD) fingers are a class of chromatin readers that recognize specific lysine residues in chromatin as a function of the methylation state, although a subset of PHD fingers can also discriminate their histone ligands based on other interactions (*e.g.*, lysine acylation, presence of arginine).^1,2^ PHD fingers are small domains consisting of ∼50–80 amino acids, typically Cys_4_-His-Cys_3_ zinc fingers, and are often found on proteins containing other PHD fingers, bromodomains, chromodomains, or Tudor domains that may contribute to multivalent histone recognition.^1,3^ In the context of PHD fingers, the specificity to the methylation state of lysine arises from the structure of the binding pocket.^4^ For example, trimethylated histone 3 lysine 4 (H3K4me3) is recognized through an aromatic cage consisting of two to four aromatic residue side chains and the trimethylammonium group is stabilized through cation-pi, hydrophobic, and van der Waals interactions. In contrast, the binding pocket of PHD fingers that preferentially bind unmodified H3K4 (H3K4me0) is rich in negatively charged residues.^1,3–5^

PHD fingers bind N-terminal tails of histone proteins to stabilize interactions of chromatin-acting proteins with chromatin and can also serve as allosteric modulators of the associated enzymes. Aberrant PHD–histone interactions, and mutations in PHD fingers, are implicated in human pathologies, including cancer and neurological and immunological disorders.^6^ PHD fingers in KDM5A,^7^ PHF23,^8,9^ and BPTF^10,11^ are fusion partners of nucleoporin-98 (NUP98) in acute myeloid leukemia. Although PHD fingers share low primary sequence similarity, they adopt a conserved globular structure that binds its histone target within a shallow binding groove. The surface-exposed binding pockets and shallow binding grooves recognize the histone ligands, which can adopt a β-sheet confirmation or helical structure to accommodate extended interactions.^7,12–14^ The nature of the binding pocket makes PHD fingers challenging targets, and difficult to engage with small molecules.^1,3,15^ This is particularly the case for PHD fingers that engage H3K4me0, which primarily rely on polar interactions for ligand binding.

There are several literature reports describing the identification of small-molecule ligands for PHD fingers. The first report utilized a HaloTag pull-down screen to identify amiodarone, a KDM5A-PHD3 inhibitor, but subsequent structure-to-activity relationship (SAR) studies revealed this class of compounds displayed weak inhibition and poor selectivity.^16,17^ A ^15^N HSQC-based fragment screen focusing on identifying ligands for the PHD finger of Pygo identified a benzimidazole derivative that binds to a cleft adjacent to the histone binding pocket.^18^ Another report utilizing an NMR-based fragment screen identified millimolar-binding fragments for the PHD fingers of BAZ2A/BAZ2B that inhibited the PHD–histone interaction.^19^ More recently, ligands that displayed low micromolar potencies for the PHD finger in UHRF1 were discovered through an AlphaScreen®-based HTS.^20^ While these examples are encouraging, the reports of small molecule ligands for PHD fingers remain rare and with low affinities for the target of interest.

**Fig. 1:**
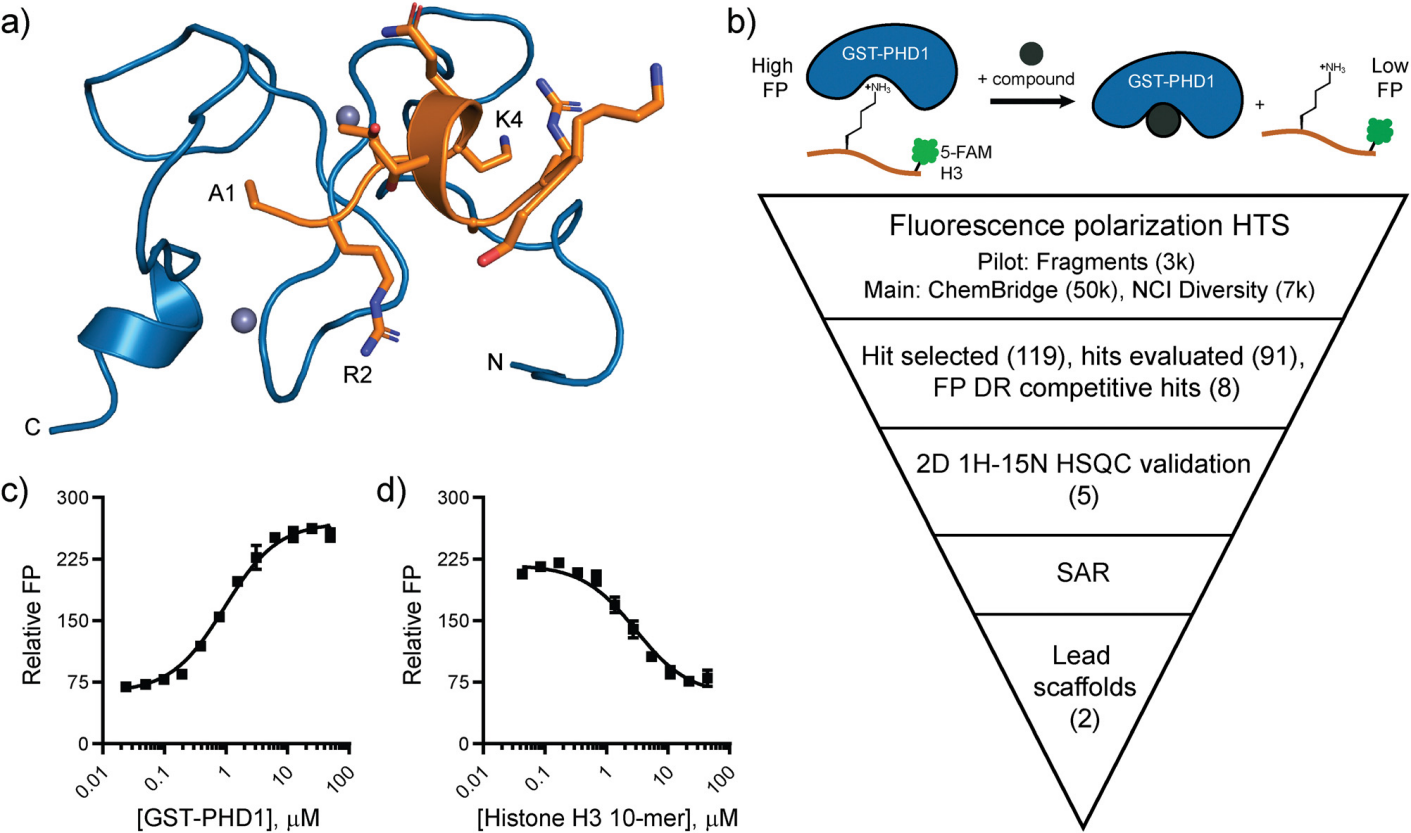
Targeting the PHD1 finger of KDM5A. (a) NMR solution structure of the PHD1 finger of KDM5A (blue) bound to histone H3 10-mer peptide (orange) (PDB: 7klr). (b) Fluorescence polarization-based high-throughput screening plan to identify ligands for PHD1. (c) Relative FP of C-terminal 5-FAM H3 10-mer peptide binding to GST-PHD1. (d) Competitive displacement of 5-FAM H3 10-mer (10 nM) binding to GST-PHD1 (2 μM) by the unlabeled H3 10-mer peptide. Error bars are ± SD for triplicate measurements.

The paucity of small molecule ligands and their low affinity for PHD fingers prompted us to investigate large-scale high-throughput screens as a strategy to discover small molecule modulators for this class of chromatin readers. We selected the PHD1 finger of histone demethylase KDM5A as the target for our investigations due to its importance in the regulation of the catalytic activity.^21,22^ KDM5A is a multi-domain enzyme with an iron-containing active site consisting of jumonji-C and jumonji-N (JmjC/N) domains, an AT-rich interactive domain (ARID) that binds DNA, a zinc-finger (Znf) domain, and three PHD fingers. Overexpression of KDM5A is associated with breast cancer proliferation and drug resistance,^23,24^ lung tumorigenesis and drug resistance,^25,26^ and gastric cancer development and progression.^27,28^ Active site inhibitors of KDM5A, such as selective, nanomolar inhibitors CPI-455,^29,30^ KDOAM-25,^31^ and KDM-C49 and its ester form C-70^32^ have found success in cancer cell models. In particular, CPI-455 was shown to reduce drug-tolerant subpopulation of cancer cells in multiple cancer cell lines, and KDOAM-25 impaired the proliferation of human myeloma cells.^30,31^ However, the competition with α-KG hampers the cellular efficacy of active site inhibitors. Our recent investigations into the regulation of catalysis in KDM5A identified PHD1 as an allosteric regulatory site in this enzyme. Specifically, binding of the product of demethylation to the PHD1 finger stimulates catalysis, enabling feed-forward regulation.^21,22^ The NMR structure of apo and bound PHD1 construct revealed binding to the N-terminus of the H3 peptide in a helical conformation, with extended hydrogen bonding and electrostatic interactions (Fig. 1a).^12^ Here, we describe the identification of PHD1 ligands through a fluorescence polarization-based high-throughput screen and several rounds of structural derivatization. ^1^H–^15^N HSQC NMR was used to validate binding and identify regions of PHD1 that interact with the ligands. The nascent SAR of the benzofuran series of ligands paves the way for further optimization.

## Results and discussion

### Design and optimization of fluorescence polarization-based assay and pilot screen

Fluorescence polarization (FP) has been widely used as a biophysical method to detect and quantify disruptions in protein–protein and protein–peptide interactions.^33,34^ It served as the primary assay in the high-throughput screening (HTS) campaign to identify ligands for the PHD1 finger (Fig. 1b). The FP assay includes a GST-labeled PHD1(S283-E344) construct and its H3K4me0 10-mer peptide substrate (Fig. 1c and d).^21,22^ GST-PHD1 binds a fluorescently labeled 5-carboxyfluorescein (5-FAM) H3 10-mer peptide with a *K*_d_ = 0.95 ± 0.08 μM (Fig. 1c). In a competitive-binding FP assay, the fluorescently labeled H3 peptide is displaced by the unlabeled H3 peptide with *K*_*i*_ = 0.95 ± 0.14 μM (Fig. 1d). The assay was miniaturized from a 100 μL volume to a 10 μL assay in low-volume 384-well plates using 50 mM HEPES pH 7.5, 50 mM KCl with 5-FAM-labeled H3 peptide (20 nM), reductant TCEP (1 mM), and 0.01% Tween-20 to avoid aggregate interference. The assay was performed using 0.5% DMSO as the negative control (0% inhibition) and excess unlabeled H3 10-mer peptide (120 μM) as the positive control (100% inhibition).

The FP assay performance in a high-throughput, automated format was first assessed by investigating the *Z* prime (*Z*′) factor, a measure reflecting the dynamic range of the assay signal and the data variation associated with measurements.^35^ Varying the concentration of GST-PHD1 (0.5, 1, 1.5, and 2 μM) in the presence or absence of 0.1% BGG revealed that the FP signal arising from the negative and positive control buffers was not affected by the presence of BGG and that 1 μM GST-PHD1 was sufficient to maintain a good dynamic range, as indicated by *Z*′ values greater than 0.5 in buffer without BGG (*Z*′ = 0.56) and with 0.1% BGG (*Z*′ = 0.59) (Fig. S1, ESI†). The pilot screen was then carried out using a fragment library of 3,095 compounds to ensure optimized assay conditions. The fragments were screened at 50 μM due to low molecular weight (median MW = 207 Da) and typical low affinities. Parallel screens were run with or without bovine gamma globulin (BGG) in the assay buffer to determine whether the hit rate would be affected by the presence of a carrier protein. The pilot screen was robust, producing an average *Z*′ = 0.72 ± 0.03 without BGG and an average *Z*′ = 0.61 ± 0.03 with BGG. Compounds that displaced the 5-FAM-labeled H3 peptide and displayed inhibition greater than or equal to 3 standard deviations above the mean were considered as hits. The pilot fragment screen without BGG produced 61 hits (1.98% hit rate). For the pilot screen using BGG, 15 hits were produced (0.5% hit rate), indicating that BGG aids in the removal of false-positive hits (Fig. S2a and b, ESI†). Pan-assay interference (PAINS), compounds that often give false-positive results due to reactivity with biological nucleophiles, redox-reactivity, photo-reactivity, metal chelators, or interfering photochromic properties,^36^ were excluded. From the 15 hits obtained through the pilot screen using BGG, one was a PAINS compound and was excluded (Fig. S2c, ESI†). The commercially available compounds (13) were repurchased, assessed for purity using LCMS, and evaluated in dose–response using the FP assay. Out of the 12 soluble compounds tested, only 7 fragments showed dose-dependent inhibition while 5 compounds displayed no measurable activity (Fig. S2d–j, ESI†).

### High-throughput screening and validation of hits

Having confirmed the robustness of the pilot screen, the screen was expanded to the ChemBridge Premium Diversity library, consisting of ∼50 000 compounds with drug-like physicochemical properties. The 50 000 compounds were tested at 10 μM concentration using the optimized assay conditions that included BGG. The screen was robust with an average *Z*′ = 0.55 ± 0.07 across 157 plates and produced 66 hits with inhibition values three standard deviations above the mean (0.13% hit rate; Fig. S3a, ESI†). Analogous to the pilot screen, PAINS compounds were excluded and 62 compounds or structurally similar derivatives (> 70% similarity) were repurchased, assessed for purity by LCMS, and tested in a dose–response format. From the 64 compounds tested, only three compounds displayed dose-dependent inhibition while the other 61 compounds displayed no measurable inhibition up to 400 μM (Fig. S3b–d, ESI†). At 400 μM, the highest concentration evaluated, 714 997, 715 958, and 727 150 inhibited the PHD1-histone interaction by 20%.

The binding of the ChemBridge screen hits to PHD1 was further evaluated using 2D ^1^H–^15^N HSQC NMR spectroscopy. This direct binding method allowed us to monitor binding by observing any ligand-induced changes in the previously assigned ^1^H–^15^N PHD1 amino acid resonances.^12^ The HSQC spectra of 50 μM ^15^N-labeled PHD1 with DMSO were overlaid with the spectra of the ^15^N-labeled PHD1 incubated with 1 mM compound. Compounds 714 997 and 727 150 did not induce any significant chemical shift perturbations (CSP) on any PHD1 resonances (data not shown). Compound 715 958 was insoluble in the NMR buffer at this concentration and was not evaluated.

**Fig. 2:**
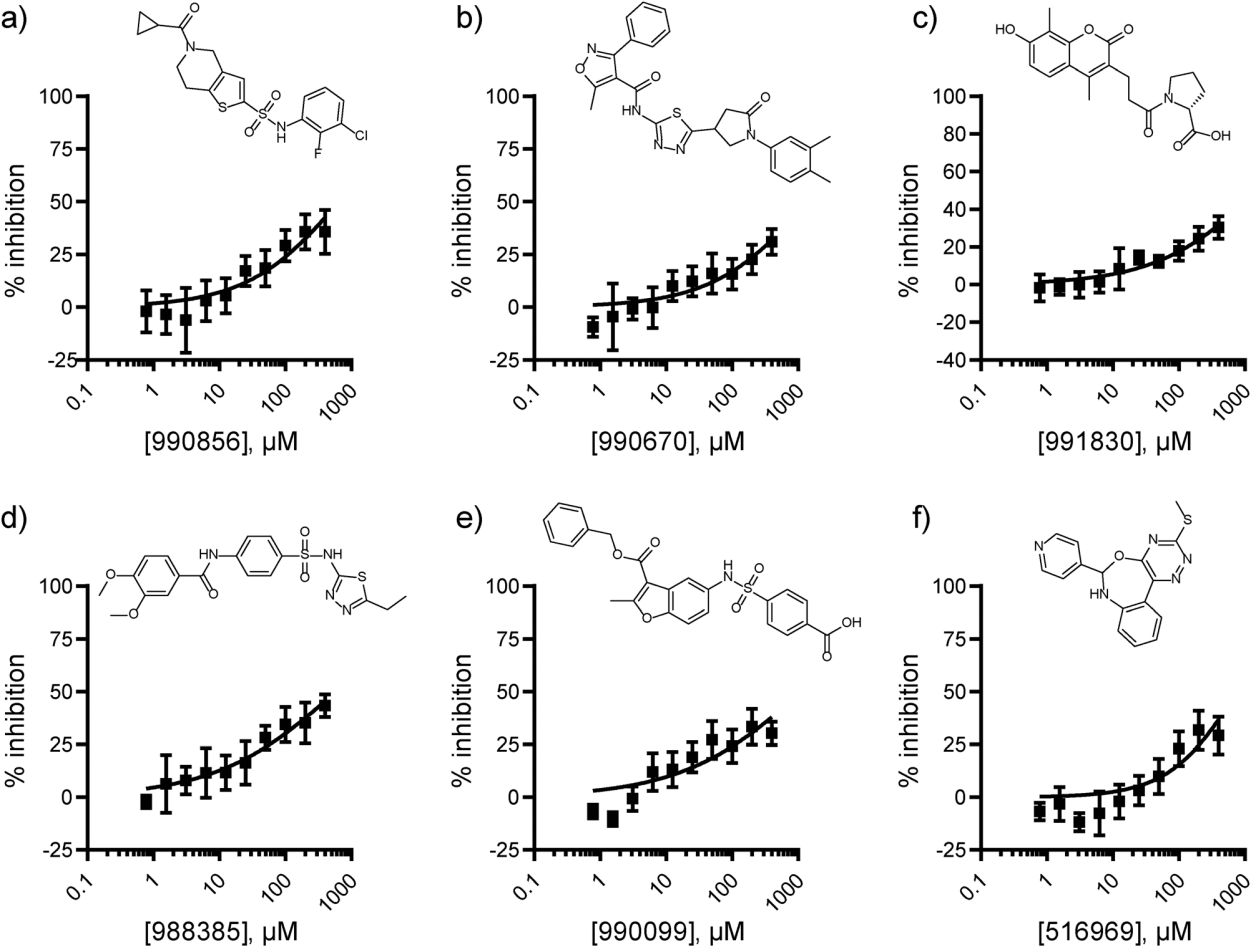
Dose-dependent inhibition of GST-PHD1 − H3 tail interaction by screen hits obtained from HTS of the NCI collection. Compounds were tested at a concentration range of 0.78–400 μM. The FP assay is carried out using 50 mM HEPES pH 7.5, 50 mM KCl buffer containing 1 μM GST-PHD1, 20 nM 5-FAM H3K4me0 10-mer peptide, 1 mM TCEP, and 0.1% BGG. DMSO is used as the negative (−) control (0% inhibition). The positive (+) control includes 120 μM unlabeled H3K4me0 10-mer (100% inhibition). Data were collected in triplicate from technical replicates and are displayed as mean ± SD.

The lack of validated hits from the ChemBridge Diversity collection screen motivated us to expand our efforts to libraries better suited for the identification of protein–protein interaction inhibitors. Specifically, the screen was expanded to include 7 027 compounds from the National Cancer Institute (NCI) collection library which encompassed protein–protein interaction inhibitors, macrocycles, and natural products (Fig. S4, ESI†). The libraries were screened at 50 μM, resulting in the identification of 38 hits with percent inhibition 3 standard deviations above the mean (0.54% hit rate; *Z*′ = 0.66 ± 0.04). Among these, the eighteen exact compounds and four structurally similar compounds that were commercially available were repurchased and evaluated by dose–response format using the FP assay. Six compounds displayed measurable dose-dependent inhibition of the PHD1-H3K4me0 interaction (Fig. 2).

The six NCI screen hits confirmed by dose response in the FP assay were further evaluated by 2D ^1^H–^15^N HSQC NMR at 1 mM to assess their direct binding to PHD1 (Fig. S5, ESI†). Compound 990 670 was insoluble in the NMR buffer and was excluded from further consideration. Out of the five compounds evaluated, 991 830, 990 856, and 988 385 caused very minor CSP on PHD1 (average shifts ≤ 0.002 ppm; Fig. S5a–c, ESI†). Conversely, 516 969, and 990 099 displayed greater average perturbations (0.0025–0.0033 ppm), while some residues were perturbed greater than 0.008 ppm (Fig. S5d and e, ESI†). The observed perturbations are low, but in the range of those induced by competitive binding fragment ligands evaluated at 5 mM with the PHD finger of BAZ2B, for which the perturbations observed were up to 0.035 ppm.^19^ Residue Cys322, one of the Zn(ii) ligands in the first zinc finger in PHD1, is the most affected by the presence of oxazepine 516 969 (Δ*δ* of 0.009 ppm; Fig. S5d, ESI†). The most prominent perturbations caused by benzofuran-containing compound 990 099 were those to residues N303, N304, D316, C322, and I324 (Fig. S5e, ESI†). Interestingly, N304 was also perturbed by aryl sulfonamide 988 385 (Δ*δ* > 0.01 ppm; Fig. S5c, ESI†). This residue is located near the first Zinc finger and is solvent-exposed.

The two molecules that caused the most prominent chemical shift perturbations, oxazepine 516 969 and benzofuran 990 099, were further evaluated. Given the complexity of the scaffold, we identified six commercially available close analogs of 516 969 to evaluate whether potency could be improved. Derivatives of 516 969 were evaluated by FP and their concentration-response curves are shown in Fig. S6 (ESI†). Compound 5 624 800 is structurally similar to NCI screen hit 516 969, but contains the pyridine nitrogen atom at an *ortho* position. It displayed an approximate IC_50_ of 0.65 mM (0.43 to 1.1 mM 95% CI), showing a modest improvement compared to 516 969 (Fig. S6b, ESI†). The phenyl analog 5 487 083 and substituted pyrazole analog 6 378 665 led to a decrease in potency (Fig. S6a and c, ESI†). Substituting the methyl group of 516 969 with ethyl in 5 620 291 also resulted in a drop in potency (Fig. S6d, ESI†).

The structural similarity between benzofuran 990 099 (Fig. 2e) and fragment hit 302 387 (Fig. S2g, ESI†) motivated us to further investigate this series. Removal of the carboxylate from 990 099 (8018–7366), drastically reduced potency, suggesting the carboxylic acid is important for PHD1 binding (Table 1 and Fig. S7a, b, ESI†). To systematically investigate how the R^3^ group on the sulfonamide substituent impacts binding, we prepared a series of derivatives where the C3 and C4 positions of the aryl ring carried various substitutions. To facilitate the rapid preparation of derivatives, the benzyl ester was replaced by a methyl ketone functionality. The synthesis of the benzofuran derivatives is outlined in Scheme S1 (ESI†) and follows a published route to obtain amino-substituted benzofuran 1.^37,38^ The route to access the benzofuran derivatives begins with a nucleophilic substitution reaction with commercially available sulfonyl chlorides and compound 1 to give substituted benzofurans B1–B7 (Scheme S1, ESI†).

**Table 1:** Competitive inhibition of GST-PHD1 by 990 099 (entry 1), analog-by-catalog derivatives (entries 2 and 4), and synthesized derivatives (entries 3, 5–10)^a^

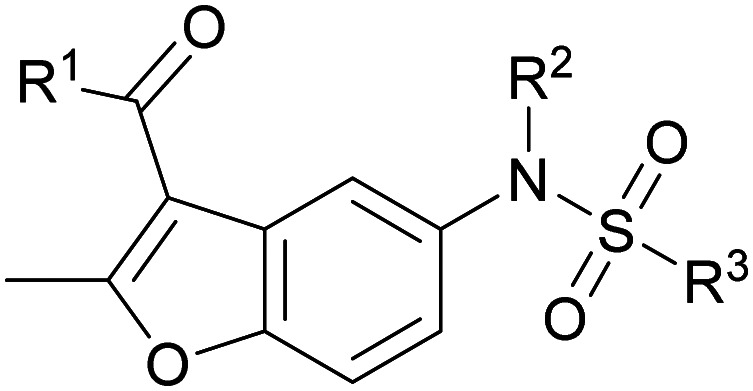					
Entry	Compound	R^1^	R^2^	R^3^	%I at 1 mM^a^
1	990 099	OBn	H	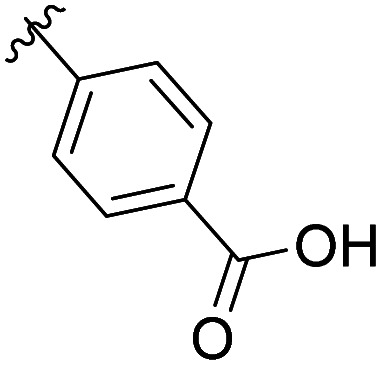	45%
2	8018-7366	OBn	H	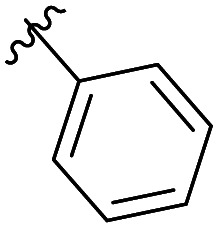	6%
3	B1	Me	H	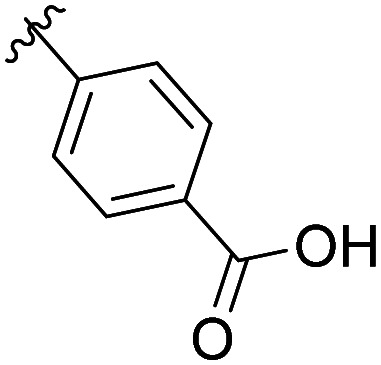	38%
4	7 917 645	Me	Ac	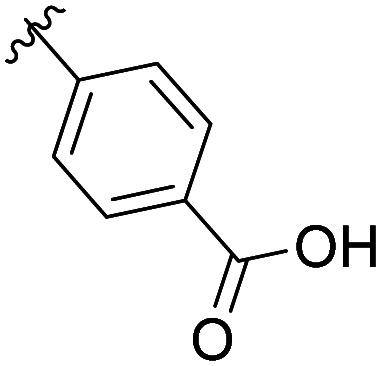	30%
5	B2	Me	H	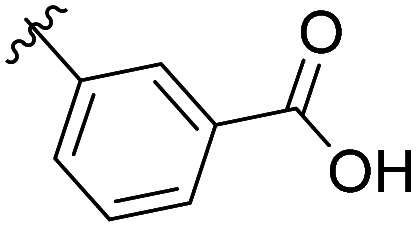	45%
6	B3	Me	H	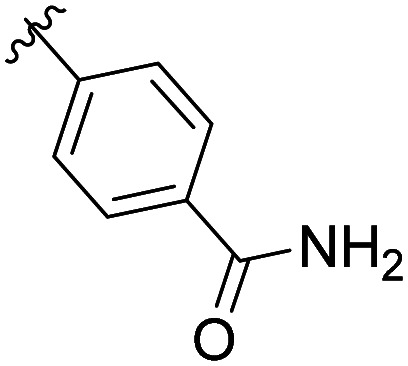	12%
7	B4	Me	H	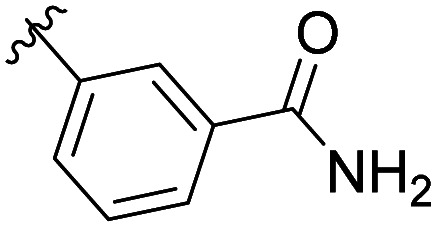	9%
8	B5	Me	H	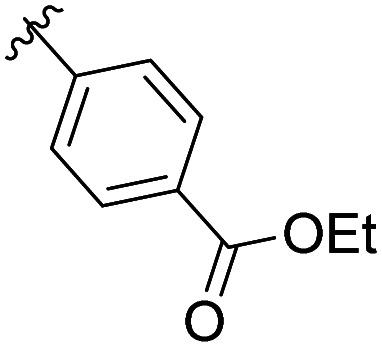	11%
9	B6	Me	H	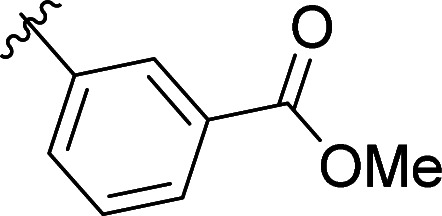	4%
10	B7	Me	H	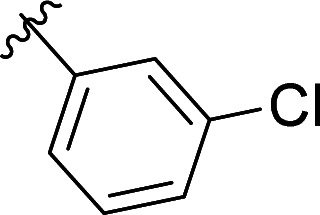	13%

a Data were collected in quadruplicate and display the % inhibition at 1 mM (*n* = 1 for R^1^ = OBn; *n* = 2 for R^1^ = Me).

The methyl ketone analog showed comparable binding affinity to the benzyl ester (Table 1, entry 1 *vs*. entry 3; Fig. S7a and c, ESI†). Capping the sulfonamide nitrogen with an acyl group in 7 917 645 (Table 1, entry 4; Fig. S7d, ESI†) slightly lowered binding affinity when compared to B1. Relative to *p*-carboxylate B1, placing the carboxylate substituent into the *meta* position on the aryl ring of B2 improved inhibition (Fig. S7c and e, ESI†). B2 displayed 45% inhibition at 1 mM, while B1 displayed 38% inhibition at 1 mM (Table 1, entries 3 *vs*. 5). Compound B2 displayed increased inhibition with an approximate IC_50_ of 1.3 mM (1.1 to 1.5 mM 95% CI) relative to 990 099, which does not reach 50% inhibition when tested up to 1.5 mM (Fig. S7a and e, ESI†). Replacing the acid with an amide (B3 and B4) or an ester (B5 and B6) also decreases the percent inhibition observed (Table 1, entries 6–9; Fig. S7f–I, ESI†). Finally, replacing the carboxylic acid in B2 with a chloro group in B7 also weakens inhibition (Table 1, entry 10; Fig. S7j, ESI†).

**Fig. 3:**
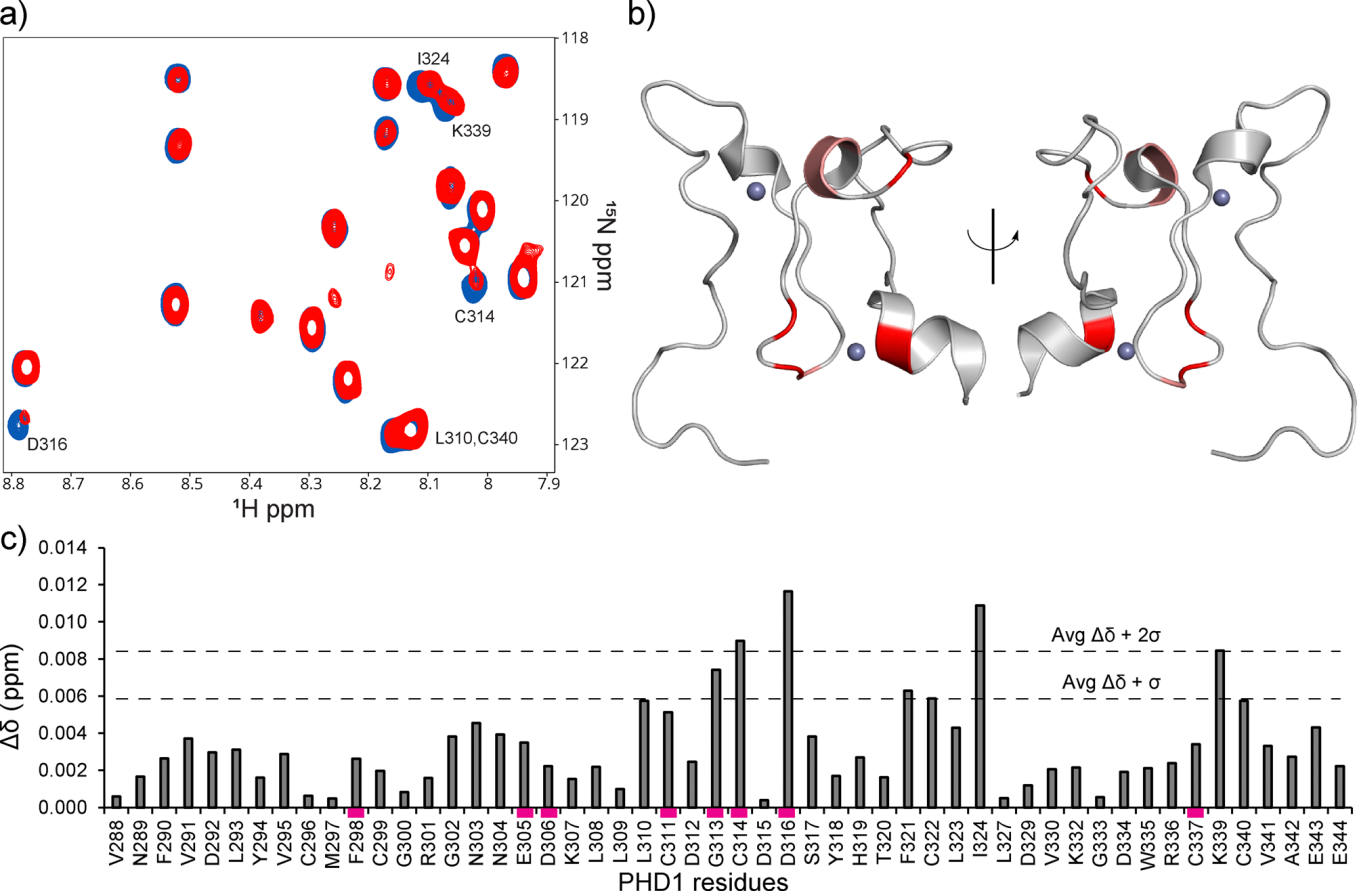
^1^H–^15^N PHD1 chemical shift perturbations induced by compound B2. (a) CSPs of PHD1 residues with 4 mM B2 (red) relative to 50 μM PHD1 with DMSO (blue). (b) CSPs induced by compound B2 that are greater than one standard deviation above the mean, indicated in panel c (PDB: 7klo). Residues colored in red display Δ*δ* + 2*σ*, while residues in salmon display Δ*δ* + *σ*. (c) CSP map of PHD1 residues affected by 4 mM compound B2 relative to PHD1 with DMSO. CSP bars highlighted by a magenta rectangle indicate residues that display broadening.

Direct binding of the identified hits to PHD1 was further probed by NMR. The binding of the best-performing synthesized compound, B2, was assessed using HSQC NMR with ^15^N PHD1. Compound B2 was tested at 0.5, 1, 2, and 4 mM with 50 μM ^15^N PHD1. As shown in Fig. 3 and Fig. S8 (ESI†), many PHD1 residue signals broadened with increasing concentration of B2, indicating intermediate exchange. While the chemical shift perturbations are small, the residues perturbed greater than one standard deviation above the mean, shown in Fig. 3, include G313, C314, D316, F321, C322, I324, and K339. Some of the residues perturbed by B2 are near the second Zn finger, including G313, C314, D316, and K339 (Fig. 1a and 3). These findings suggest that compound B2 may be interacting with residues in this region or interacting with Zn directly.

## Conclusions

In summary, we describe the first HTS campaign aimed at identifying ligands for the PHD1 finger of KDM5A. Screen hits were confirmed by fluorescence polarization dose–response binding assays and ^1^H–^15^N HSQC NMR, which led us to investigate and explore SAR around two hit scaffolds, oxazepanes and benzofurans. For the benzofuran series, this included synthesizing derivatives B1–B7, which simplified the scaffold for ease of synthesis through the replacement of the benzyl ester with a methyl ketone group and allowed us to explore derivatization around the sulfonamide substituent. Compound B2 inhibits the PHD1-histone interaction and causes chemical shift perturbations on PHD1 amide backbone resonances in ^1^H–^15^N HSQC experiments, indicative of direct binding. Our findings are consistent with challenges associated with targeting shallow solvent-exposed binding sites of PHD fingers that recognize unmodified H3K4, as evident by the single account reporting competitive binding fragments for the BAZ2 PHD fingers.^19^ The oxazepane and benzofuran competitive ligands identified here will serve as the starting point for future optimization efforts.

## Conflicts of interest

The authors declare that they have no known competing financial interests or personal relationships that could have appeared to influence the work reported in this paper.

## Supplementary Material

CB-005-D3CB00214D-s001
