# Supplementary material for: Identifying ligands for the PHD1 finger of KDM5A through high-throughput screening
Source: RSC Chem Biol. 2023 Dec 18;5(3):209–15. doi: 10.1039/d3cb00214d (PMC10915964; doi:10.1039/d3cb00214d)
Supplement: CB-005-D3CB00214D-s001 [file CB-005-D3CB00214D-s001.pdf]

Electronic Supplementary Material (ESI) for RSC Chemical Biology.
This journal is © The Royal Society of Chemistry 2023

Supporting Information for:

Identifying ligands for the PHD1 finger of KDM5A through high-throughput screening

Authors

Gloria Ortiz[1], James E. Longbotham[1], Sophia Qin[1], Meng Yao Zhang[1], Gregory M. Lee[2,3], R. Jeffrey Neitz[2,3], Mark J. S. Kelly[3], Michelle Arkin[2,3], Danica Galonić Fujimori[1,3,4,*]

Affiliations

[1]Department of Cellular and Molecular Pharmacology, University of California San Francisco; San Francisco, CA 94158, USA.

[2]Small Molecule Discovery Center (SMDC), University of California San Francisco; San Francisco, CA 94158, USA.

[3]Department of Pharmaceutical Chemistry, University of California San Francisco; San Francisco, CA 94158, USA.

[4]Quantitative Biosciences Institute (QBI), University of California San Francisco; San Francisco, CA 94158, USA.

*Correspondence: danica.fujimori@ucsf.edu

Table of Contents

Reagents and materials 2

Chemical synthesis and characterization 3

Expression of GST-PHD1(S287-E344) and GST tag removal 3

Fluorescence polarization high-throughput screening 4

Nuclear magnetic resonance (NMR) binding assays 5

Synthetic Procedures 6

4-(N-(3-acetyl-2-methylbenzofuran-5-yl)sulfamoyl)benzoic acid (**B1**): 6

3-(N-(3-acetyl-2-methylbenzofuran-5-yl)sulfamoyl)benzoic acid (**B2**): 6

4-(N-(3-acetyl-2-methylbenzofuran-5-yl)sulfamoyl)benzamide (**B3**): 7

3-(N-(3-acetyl-2-methylbenzofuran-5-yl)sulfamoyl)benzamide (**B4**): 7

ethyl 4-(N-(3-acetyl-2-methylbenzofuran-5-yl)sulfamoyl)benzoate (**B5**): 8

methyl 3-(N-(3-acetyl-2-methylbenzofuran-5-yl)sulfamoyl)benzoate (**B6**): 8

N-(3-acetyl-2-methylbenzofuran-5-yl)-3-chlorobenzenesulfonamide (**B7**): 9

Supplementary Figures and Schemes 10

Scheme S1. 10

Figure S1. 11

Figure S2. 12
Figure S3. 13
Figure S4. 14
Figure S5. 15
Figure S6. 16
Figure S7. 17
Figure S8. 18
Supporting Spectra 19
Spectrum S1. ^{1}H NMR of B1 19
Spectrum S2. ^{13}C NMR of B1 19
Spectrum S3. ^{1}H NMR of B2 20
Spectrum S4. ^{13}C NMR of B2 20
Spectrum S5. ^{1}H NMR of B3 21
Spectrum S6. ^{13}C NMR of B3 21
Spectrum S7. ^{1}H NMR of B4 22
Spectrum S8. ^{13}C NMR of B4 22
Spectrum S9. ^{1}H NMR of B5 23
Spectrum S10. ^{13}C NMR of B5 23
Spectrum S11. ^{1}H NMR of B6 24
Spectrum S12. ^{13}C NMR of B6 24
Spectrum S13. ^{1}H NMR of B7 25
Spectrum S14. ^{13}C NMR of B7 25
Supplementary references 26

Reagents and materials

The Small Molecule Discovery Center (SMDC) Fragment library includes 3,095 compounds (Life Chemicals, Munich, Germany; Maybridge, Loughborough, UK; and Asinex, Winston-Salem, NC). The ChemBridge Premium Diversity library (ChemBridge, San Diego, CA) containing 50,000 compounds and NCI Diversity collection of macrocycles (291 compounds), natural products (872 compounds), and protein-protein inhibitors (6,044 compounds) were used for high-throughput screening. The ChemBridge Premium Diversity collection contains compounds with drug-like physicochemical properties and a diverse set of small molecules. Fragment screen hits selected for follow-up investigations were purchased from Life Chemicals, Vitas-M, MayBridge, ChemDiv, MayBridge, Alfa-Aesar, FisherSci, Enamine, Combi-Blocks, and Toronto Research Chemicals. ChemBridge screen hits were obtained from ChemBridge. NCI screen hits were purchased from Vitas-M, Life Chemicals, and ChemDiv. The catalog numbers for repurchased compounds are the compound identifiers in the manuscript. Buffer reagents and additives were purchased from Sigma-Aldrich.

Chemical synthesis and characterization

Chemical reagents and solvents (dry) used for synthesis were purchased from commercial suppliers and used without further purification. Previously synthesized 1-(5-amino-2-methylbenzofuran-3-yl)ethan-1-one was prepared according to the literature procedures.[1,2] All reactions were carried out in oven-dried flasks sealed with septa and conducted under a nitrogen atmosphere unless otherwise noted. Silica gel thin layer chromatography (TLC; 250 μM) was performed on precoated TLC glass plates and visualized under UV light. Flash column chromatography was performed on Silicycle Silica Flash F60 (230-400 Mesh) using a forced flow of air at 0.5-1.0 bar. NMR spectra were recorded on a Bruker Avance Neo-600 MHz spectrometer. Chemical shifts (δ) are expressed in parts per million (ppm) and are referenced to DMSO (2.50 ppm, 40 ppm). Coupling constants are reported as Hertz (Hz). Splitting patterns are indicated as follows: s, singlet; d, doublet; t, triplet; q, quartet; dd, doublet of doublet; m, multiplet. Preparative high performance liquid chromatography (HPLC) was performed on a Waters Prep 150Q LC system with a Waters 2998 Photodiode Array detector, and this system was operated using ChromScope software. All separations used linear gradients of water containing 0.1% trifluoroacetic acid and acetonitrile, at a flow rate of 10 mL/min (XSelect® Peptide CSH™ C18, OBD™ Prep Column, 130Å, 5 μM, 19 mm × 250 mm). Reactions were monitored using a Waters Acquity UPLC (BEH, C18, 1.7 μm column) and eluted with a linear gradient of 5–95% acetonitrile/water (+0.1% formic acid) over 3.0 min. Chromatograms were recorded with a UV detector set at 254 nm and a time-of-flight mass spectrometer (Waters Xevo G2-XS).

Expression of GST-PHD1(S287-E344) and GST tag removal

The GST-PHD1(S287-E344) construct was expressed and purified as previously described.[3,4] Briefly, GST-PHD1(S287-E344) was cloned into a pET41a vector and expressed in BL21 (DE3) E. coli cells. Cells were induced with 0.4 mM IPTG in LB broth supplemented with 50 μM $ZnCl_2$ and grown overnight at 18 °C before the pellet was collected. The cells were resuspended in lysis buffer consisting of 140 mM NaCl, 2.7 mM KCl, 10 mM Na_2HPO_4, 1.8 mM KH_2PO_4, 5 mM BME, 50 μM $ZnCl_2$, 1 mM phenylmethylsulfonyl fluoride (PMSF), pH 7.3. The cells were lysed by sonication and centrifuged. The supernatant was purified using Glutathione Sepharose 4B resin, washed with high salt buffer (50 mM HEPES, 700 mM KCl, 10% glycerol, 5 mM BME, 50 μM $ZnCl_2$, 1 mM PMSF, pH 8.0) and eluted using low salt buffer with glutathione (50 mM HEPES, 150 mM KCl, 10% glycerol, 5 mM BME, 50 μM $ZnCl_2$, 1 mM PMSF, pH 8.0 with 30 mM glutathione). The sample was then concentrated and further purified by size-exclusion chromatography using a Hiload 26/60 Superdex 75 gel filtration column. The sample was eluted into 40 mM HEPES pH 8.0, 50 mM KCl, 5 mM BME, 50 μM $ZnCl_2$, and flash-frozen.

The GST tag from GST-PHD1(S287-E344) was cleaved from the sample using TEV protease. The reaction was run using 40 mM HEPES pH 8.0 with 50 mM KCl, 5 mM BME, and 50 μM $ZnCl_2$ at 4 °C and a 30 mL Thermo Slide-A-lyzer G2 dialysis cassette (3.5 K MW co) overnight. The sample was collected and concentrated. To remove excess His-tagged TEV protease, a Ni-NTA resin purification was performed using 40 mM HEPES pH 8.0 buffer with 50 mM KCl, 2.5 mM BME, and 50 μM $ZnCl_2$. The flow through fractions were collected and BME was restored to 5 mM. The GST tag was removed by using Glutathione Sepharose 4B resin, washed with high salt buffer (50 mM HEPES, 700 mM KCl, 10% glycerol, 5 mM BME, 50 μM $ZnCl_2$, 1 mM PMSF, pH 8.0) and eluted using low salt buffer with glutathione (50 mM HEPES, 150 mM KCl, 10% glycerol, 5 mM BME, 50 μM $ZnCl_2$, 1 mM PMSF, pH 8.0 with 30 mM glutathione). The flow-through sample was then concentrated and further purified by size-exclusion chromatography using a Hiload 26/60 Superdex 75 gel filtration column. The sample was eluted into 40 mM buffer containing HEPES pH 8.0, 50 mM KCl, 5 mM BME, and 50 μM $ZnCl_2$ and flash-frozen.

Fluorescence polarization high-throughput screening

To test the activity of purified GST-PHD1(S287-E344), direct-binding and competitive-binding fluorescence polarization (FP) were done, as previously described.[3,4] For screening, GST-PHD1(S287-E344) was diluted in buffer (50 mM HEPES, pH 7.5, 50 mM KCl, 0.01% Tween-20, 1 mM TCEP) with 20 nM 5-carboxyfluorescein-labeled H3 10-mer peptide and with or without 0.1% bovine gamma globulin (BGG). This was treated as the assay buffer (with 0.1% BGG) and also as the negative control. For the positive control, 120 µM unlabeled H3K4 10-mer peptide was added to the assay buffer. The assay buffer was dispensed using the EL406 Biotek liquid dispenser onto black, round-bottom low volumes 384-microwell plates (Corning 4514) to a final volume of 10 µL per well. DMSO (10 nL or 50 nL) or screening compounds at 10 nL (ChemBridge Premium Diversity collection at 10 µM final) or 50 nL (ML fragments and NCI libraries at 50 µM final) was added to plates containing the assay buffer using the BioMek liquid handler/dispenser. The final DMSO in the samples was either 0.1% or 0.5%. Fluorescence polarization measurements were performed directly and after 45 minutes of incubation at room temperature using a Perkin Elmer Envision and XCite 2105 multimode microplate reader equipped with a stacker. Excitation light was delivered with a xenon flash lamp at 480/30 nm (BW) and emission was collected after passing through an emission filter (535/25 nm). For fluorescence polarization experiments, two 535/40 nm filters (S, P) and dual dichroic D505/D535 nm mirrors were used.

For FP-based assay optimization, the assay signal dynamic range was evaluated using the negative and positive control buffers with varying amounts of GST-PHD1 (0.5, 1, 1.5, or 2 µM) in the presence or absence of 0.1% BGG. For each condition, buffer was dispensed into 3 columns of a 384-well plate (48 wells each) using the BioMek liquid dispenser and the FP signal was recorded. The Z′ value reports on the assay robustness by reflecting both the dynamic range and the data variation associated with assay measurements.[5] The Z′ value is given by the following equation:

$$Z' = 1 - \frac{3\sigma_{PC} + 3\sigma_{NC}}{|\mu_{PC} - \mu_{NC}|}$$

where σ represents the standard deviation, PC represents the positive control, NC represents the negative control, and µ represents the mean value of the FP signal.

Screen results were analyzed using the UCSF SMDC web application called HiTS (http://smdc.ucsf.edu/hits/). Screen hits represent compounds that displayed inhibition greater than or equal to 3 standard deviations of the mean percent inhibition values, where the negative control is set to 0% inhibition and the positive control is set to 100% inhibition. Cheminformatics analysis on the screen hits was done using DataWarrior, an open-source program for data visualization and analysis with chemical intelligence.[6] The Tanimoto coefficient was used to gauge the similarity and diversity of screen hits and cluster any compounds with greater than 75% similarity.

Screen hits were repurchased when available. Structurally similar derivatives were purchased when exact compounds were not available for purchase. Compound purity was confirmed by LCMS. All compounds were dissolved in DMSO-d_6 to a final concentration of 40 mM unless otherwise specified. All putative screen hits were confirmed in dose-response using the competitive binding fluorescence polarization assay. All compound stock plates were prepared in 10-point, 2-fold serial dilutions starting from the stock concentration of 40 mM. Initial compounds were pinned using either a Biomek liquid handler (2 x 50 nL pins) for a concentration series of 0-400 µM or using the Echo liquid handler for a concentration series of 0-800 µM (2% DMSO final). The benzofuran series of compounds were dissolved in DMSO-d_6 to a final concentration of 50 or 100 mM and dispensed using the Echo liquid dispenser for a concentration series of 2.9-1500 µM (from 50 mM, 3.7% DMSO) or 3.9-200 µM (from 100 mM, 2% DMSO). Data analysis for

dose-response inhibition experiments was done using GraphPad Prism 9.5 (GraphPad Software Inc., San Diego, CA). Data were normalized to the negative control (0% inhibition) and the positive control (100% inhibition). Curves were fit using the Dose-response – inhibition log(inhibitor) vs. response – variable slope equation in GraphPad to generate the IC_{50} values or approximate IC_{50} values. All dose-response experiments were conducted in triplicate or quadruplicate and are reported as the mean ± SD, unless otherwise noted.

Nuclear magnetic resonance (NMR) binding assays

A PHD1(S287-E344) construct was used in NMR studies, as previously described.[4] Briefly, the GST-PHD1 fusion construct was expressed in M9 minimal media containing ^{15}N ammonium chloride. The labeled GST-PHD1 was purified and the GST tag was cleaved as described in the Expression of GST-PHD1(S287-E344) and GST tag removal section. For 2D 1H, ^{15}N Fast HSQC (FHSQC) binding experiments, 50 µM ^{15}N-labeled PHD1 was used and spectra were measured after the addition of DMSO-d_6 or compound at 1 mM in DMSO-d_6 for single concentration experiments. The NMR samples were prepared in 50 mM HEPES pH 7.5, 150 mM KCl buffer with 5 mM BME, 100 µM $ZnCl_2$, and 10% D_2O at a final concentration of 2.4% DMSO-d_6. The HSQC spectra were collected on a 600 MHz Bruker Avance NEO spectrometer equipped with an actively shielded Z-gradient 5 mM TCI cryoprobe using programs from the pulse program library (TopSpin 4.0.6). FHSQC acquisition parameters were: NS = 8, acquisition time = 0.106 s ^1H, 0.060 s ^{15}N. All experiments were carried out at 298 K (calibrated with 4% v/v MeOH in MeOD).

For 2D ^1H, ^{15}N FHSQC titration experiments, 50 µM ^{15}N-labeled PHD1 was incubated with 0, 0.5, 1, 2, or 4 mM B2 sample with a final concentration of 2.9% DMSO-d_6 for all samples. The NMR samples were prepared in 50 mM HEPES pH 7.5, 150 mM KCl buffer with 5 mM BME, 100 µM $ZnCl_2$, and 5% D_2O. The 2D ^{15}N FHSQC spectra for the titration series were recorded at 298 K on a Bruker 800 MHz AVANCE-I spectrometer equipped with an actively shielded Z-gradient TCI cryoprobe using programs from the pulse program library (TopSpin 4.0.8). FHSQC acquisition parameters were: NS = 8, acquisition time = 0.082 s ^1H, 0.045s ^{15}N. The analysis of the chemical shift perturbation of the amide cross-peaks during the titration with the ligand was performed using CCPNMR Analysis software and use PHD1 with 2.9% DMSO-d_6 as the reference.[7] Apo PHD1 backbone assignments were imported from the BMRB (entry 30808).[4] The chemical shift perturbations for all the NMR experiments were calculated in CCPNMR v3.2, which uses the following equation:

$$\Delta\, chemical\ shift = \sqrt{\Delta H^2 + 0.15(\Delta N)^2}$$

Synthetic Procedures

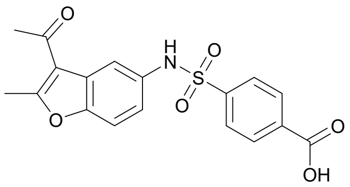

4-(N-(3-acetyl-2-methylbenzofuran-5-yl)sulfamoyl)benzoic acid (**B1**):

To a solution of 1-(5-amino-2-methylbenzofuran-3-yl)ethan-1-one (80 mg, 0.42 mmol, 1.0 eq) and 4-chlorosulfonyl benzoic acid (103 mg, 0.46 mmol, 1.1 eq) in 3.0 mL acetone and 1.5 mL DMF was added sodium bicarbonate solution (0.28 M in H_2O, 1.0 mL, 0.66 eq). The mixture was stirred overnight at RT. The next day, the acetone was evaporated under reduced pressure. To the residual mixture was added 2 mL 1 M HCl and stirred for 1 h and the residual mixture was filtered and washed with water (2 x 10 mL). The resulting crude residue was purified by preparative HPLC to yield **B1** (60 mg, 38%) as a white solid.

^{1}H NMR (600 MHz, DMSO-d_6) δ 10.35 (s, 1H), 8.04 (d, J = 8.1 Hz, 2H), 7.81 (d, J = 8.1 Hz, 2H), 7.76 (s, 1H), 7.47 (d, J = 8.7 Hz, 1H), 7.05 (dd, J = 8.8, 2.1 Hz, 1H), 2.74 (s, 3H), 2.51 (s, 3H).

^{13}C NMR (151 MHz, DMSO-d_6) δ 194.0, 166.6, 164.4, 150.6, 143.3, 134.9, 134.0, 130.5, 127.5, 127.0, 119.5, 117.5, 115.2, 111.8, 40.4, 40.3, 40.1, 40.0, 39.8, 39.7, 39.6, 31.1, 15.8.

HRMS: m/z calcd. for $C_{18}H_{16}NO_6S^+$ ($[M+H]^+$): 374.0693, found 374.0705.

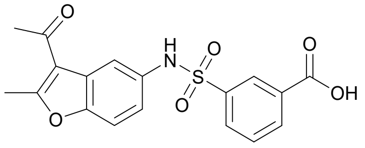

3-(N-(3-acetyl-2-methylbenzofuran-5-yl)sulfamoyl)benzoic acid (**B2**):

To a solution of 1-(5-amino-2-methylbenzofuran-3-yl)ethan-1-one (80 mg, 0.42 mmol, 1.0 eq) and 3-(chlorosulfonyl)benzoic acid (103 mg, 0.46 mmol, 1.1 eq) in 3.0 mL acetone and 1.5 mL DMF was added sodium bicarbonate solution (0.28 M in H_2O, 1.0 mL, 0.66 eq). The reaction was stirred overnight at RT. The next day, the acetone was evaporated under reduced pressure and the residual mixture was portioned between 25 mL of 1 M HCl and 50 mL EtOAC. The organics were extracted and the aqueous phase washed with EtOAc (2 x 25 mL). The combined organic extracts were washed with water and brine, dried over sodium sulfate, and concentrated under reduced pressure. The resulting crude residue was purified by preparative HPLC to yield **B2** (99 mg, 63%) as a white solid.

^{1}H NMR (600 MHz, DMSO-d_6) δ 13.46 (s, 1H), 10.31 (s, 1H), 8.29 (s, 1H), 8.11 (d, J = 7.7 Hz, 1H), 7.89 (d, J = 7.8 Hz, 1H), 7.75 (s, 1H), 7.65 (t, J = 7.8 Hz, 1H), 7.48 (d, J = 8.8 Hz, 1H), 7.04 (d, J = 8.8 Hz, 1H), 2.74 (s, 3H), 2.51 (s, 3H).

^{13}C NMR (151 MHz, DMSO-d_6) δ 193.9, 166.4, 164.4, 150.6, 140.2, 134.0, 133.8, 132.2, 131.2, 130.3, 127.9, 126.9, 119.6, 117.5, 115.2, 111.8, 40.4, 40.3, 40.1, 40.0, 39.9, 39.7, 39.6, 31.1, 15.8.

HRMS: m/z calcd. for $C_{18}H_{16}NO_6S^+$ ($[M+H]^+$): 374.0693, found 374.0705.

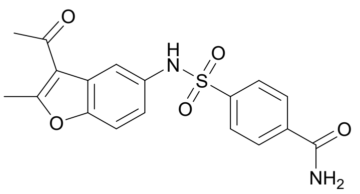

4-(N-(3-acetyl-2-methylbenzofuran-5-yl)sulfamoyl)benzamide (**B3**):

To a solution of 1-(5-amino-2-methylbenzofuran-3-yl)ethan-1-one (60 mg, 0.32 mmol, 1.0 eq) and 4-(chlorosulfonyl)benzamide (77 mg, 0.35 mmol, 1.1 eq) in 2.2 mL acetone and 1.1 mL DMF was added sodium bicarbonate solution (0.28 M in H_2O, 0.75 mL, 0.66 eq). The mixture was stirred overnight at RT. The next day, the acetone was evaporated under reduced pressure. To the residual mixture was added 2 mL 1 M HCl and stirred for 1 h and the residual mixture was filtered and washed with water (2 x 10 mL). The resulting crude residue was purified by preparative HPLC to yield **B3** (59 mg, 50%) as a white solid.

^{1}H NMR (600 MHz, DMSO-d_6) δ 10.31 (s, 1H), 8.10 (s, 1H), 7.95 (d, J = 8.1 Hz, 2H), 7.80 – 7.75 (m, 3H), 7.57 (s, 1H), 7.47 (d, J = 8.7 Hz, 1H), 7.06 (dd, J = 8.8, 2.0 Hz, 1H), 2.74 (s, 3H), 2.51 (s, 3H).

^{13}C NMR (151 MHz, DMSO-d_6) δ 194.0, 167.1, 164.4, 150.5, 141.9, 138.5, 134.1, 128.7, 127.2, 126.9, 119.3, 117.5, 114.9, 111.8, 40.4, 40.3, 40.1, 40.0, 39.8, 39.7, 39.6, 31.1, 15.8.

HRMS: m/z calcd. for $C_{18}H_{17}N_2O_5S^+$ ($[M+H]^+$): 373.0853, found 373.0864.

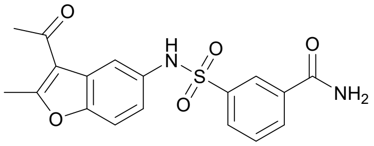

3-(N-(3-acetyl-2-methylbenzofuran-5-yl)sulfamoyl)benzamide (**B4**):

To a solution of 1-(5-amino-2-methylbenzofuran-3-yl)ethan-1-one (79 mg, 0.42 mmol, 1.0 eq) and 3-carbamoylbenzenesulfonyl chloride (101 mg, 0.46 mmol, 1.1 eq) in 2.9 mL acetone and 1.5 mL DMF was added sodium bicarbonate solution (0.28 M in H_2O, 1.0 mL, 0.66 eq). The reaction was stirred overnight at RT. The next day, the acetone was evaporated under reduced pressure and the residual mixture was portioned between 25 mL of 1 M HCl and 50 mL EtOAC. The organics were extracted and the aqueous phase washed with EtOAc (2 x 25 mL). The combined organic extracts were washed with water and brine, dried over sodium sulfate, and concentrated under reduced pressure. The resulting crude residue was purified by preparative HPLC to yield **B4** (54 mg, 63%) as a white solid.

^{1}H NMR (600 MHz, DMSO-d_6) δ 10.27 (s, 1H), 8.26 (s, 1H), 8.16 (s, 1H), 8.05 (d, J = 7.7 Hz, 1H), 7.82 (d, J = 7.8 Hz, 1H), 7.75 (s, 1H), 7.61 (t, J = 7.9 Hz, 1H), 7.57 (s, 1H), 7.47 (d, J = 8.8 Hz, 1H), 7.05 (d, J = 8.7 Hz, 1H), 2.74 (s, 3H), 2.51 (s, 3H).

^{13}C NMR (151 MHz, DMSO-d_6) δ 194.0, 166.8, 164.4, 150.5, 140.1, 135.6, 134.2, 131.8, 129.8, 129.7, 126.9, 126.7, 119.4, 117.5, 115.0, 111.8, 40.4, 40.3, 40.1, 40.0, 39.8, 39.7, 39.6, 31.1, 15.8.

HRMS: m/z calcd. for $C_{18}H_{17}N_2O_5S^+$ ($[M+H]^+$): 373.0853, found 373.0864.

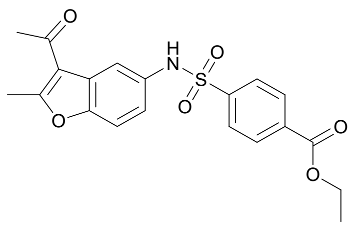

ethyl 4-(N-(3-acetyl-2-methylbenzofuran-5-yl)sulfamoyl)benzoate (**B5**):

To a solution of 1-(5-amino-2-methylbenzofuran-3-yl)ethan-1-one (60 mg, 0.42 mmol, 1.0 eq) and ethyl 4-(chlorosulfonyl)benzoate (116 mg, 0.46 mmol, 1.1 eq) in 3.0 mL acetone and 1.5 mL DMF was added sodium bicarbonate solution (0.28 M in H_2O, 1.0 mL, 0.66 eq). The mixture was stirred overnight at RT. The next day, the acetone was evaporated under reduced pressure. To the residual mixture was added 2 mL 1 M HCl and stirred for 1 h and the residual mixture was filtered and washed with water (2 x 10 mL). The resulting crude residue was purified by preparative HPLC to yield **B5** (113 mg, 66%) as a white solid.

^{1}H NMR (600 MHz, DMSO-d_6) δ 10.37 (s, 1H), 8.06 (d, J = 8.2 Hz, 2H), 7.83 (d, J = 8.2 Hz, 2H), 7.76 (s, 1H), 7.47 (d, J = 8.7 Hz, 1H), 7.04 (dd, J = 8.8, 2.0 Hz, 1H), 4.31 (q, J = 7.1 Hz, 2H), 2.74 (s, 3H), 2.50 (s, 3H), 1.30 (t, J = 7.1 Hz, 3H).

^{13}C NMR (151 MHz, DMSO-d_6) δ 194.0, 165.0, 164.4, 150.6, 143.6, 133.9, 130.4, 127.7, 127.0, 119.6, 117.5, 115.3, 111.8, 61.8, 40.4, 40.3, 40.1, 40.0, 39.8, 39.7, 39.6, 31.1, 15.8, 14.5.

HRMS: m/z calcd. for $C_{20}H_{20}NO_6S^+$ ($[M+H]^+$): 402.1006, found 402.0979.

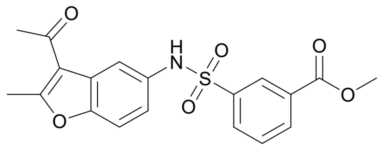

methyl 3-(N-(3-acetyl-2-methylbenzofuran-5-yl)sulfamoyl)benzoate (**B6**):

To a solution of 1-(5-amino-2-methylbenzofuran-3-yl)ethan-1-one (80 mg, 0.42 mmol, 1.0 eq) and methyl 3-(chlorosulfonyl)benzoate (129 mg, 0.55 mmol, 1.3 eq) in 2.2 mL acetone was added sodium bicarbonate solution (1.76 M in H_2O, 0.72 mL, 3.0 eq). The reaction was stirred overnight at RT. The next day, the acetone was evaporated under reduced pressure and the residual mixture was portioned between 25 mL of 1 M HCl and 50 mL EtOAC. The organics were extracted and the aqueous phase washed with 1 M HCl (2 x 25 mL). The combined organic extracts were washed with water and brine, dried over sodium sulfate, and concentrated under reduced pressure. The resulting crude residue was purified by preparative HPLC to yield **B6** (128 mg, 78%) as a white solid.

^{1}H NMR (600 MHz, DMSO-d_6) δ 10.35 (s, 1H), 8.30 (s, 1H), 8.14 (d, J = 7.7 Hz, 1H), 7.92 (d, J = 7.8 Hz, 1H), 7.76 (s, 1H), 7.68 (t, J = 7.9 Hz, 1H), 7.48 (d, J = 8.8 Hz, 1H), 7.04 (d, J = 8.7 Hz, 1H), 3.87 (s, 3H), 2.74 (s, 3H), 2.52 (s, 3H).

^{13}C NMR (151 MHz, DMSO-d_6) δ 194.0, 165.3, 164.4, 150.6, 140.4, 134.0, 133.7, 131.6, 131.0, 130.5, 127.7, 127.0, 119.6, 117.5, 115.2, 111.8, 53.1, 40.4, 40.3, 40.1, 40.0, 39.8, 39.7, 39.6, 31.1, 15.8.

HRMS: m/z calcd. for $C_{19}H_{18}NO_6S^+$ ($[M+H]^+$): 388.0849, found 388.0830.

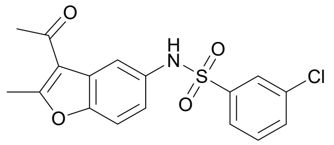

N-(3-acetyl-2-methylbenzofuran-5-yl)-3-chlorobenzenesulfonamide (**B7**):

To a solution of 1-(5-amino-2-methylbenzofuran-3-yl)ethan-1-one (80 mg, 0.42 mmol, 1.0 eq) and 3-chlorobenzenesulfonyl chloride (116 mg, 0.55 mmol, 1.3 eq) in 2.2 mL acetone was added sodium bicarbonate solution (1.76 M in H_2O, 0.72 mL, 3.0 eq). The reaction was stirred overnight at RT. The next day, the acetone was evaporated under reduced pressure and the residual mixture was portioned between 25 mL of 1 M HCl and 50 mL EtOAC. The organics were extracted and the aqueous phase washed with 1 M HCl (2 x 25 mL). The combined organic extracts were washed with water and brine, dried over sodium sulfate, and concentrated under reduced pressure. The resulting crude residue was purified by preparative HPLC to yield **B7** (118 mg, 77%) as a white solid.

^{1}H NMR (600 MHz, DMSO-d_6) δ 10.31 (s, 1H), 7.76 (s, 1H), 7.73 (s, 1H), 7.69 (d, J = 7.9 Hz, 1H), 7.63 (d, J = 7.8 Hz, 1H), 7.56 (t, J = 7.8 Hz, 1H), 7.50 (d, J = 8.7 Hz, 1H), 7.05 (d, J = 8.8 Hz, 1H), 2.75 (d, J = 1.8 Hz, 3H), 2.53 (s, 3H).

^{13}C NMR (151 MHz, DMSO-d_6) δ 194.0, 164.5, 150.6, 141.5, 134.2, 133.9, 133.3, 131.8, 127.0, 126.7, 125.9, 119.6, 117.5, 115.3, 111.8, 40.4, 40.3, 40.1, 40.0, 39.8, 39.7, 39.6, 31.1, 15.8, 1.63.

HRMS: m/z calcd. for $C_{17}H_{15}ClNO_4S^+$ ($[M+H]^+$): 364.0405, found 364.0403.

Supplementary Figures and Schemes

Scheme S1.

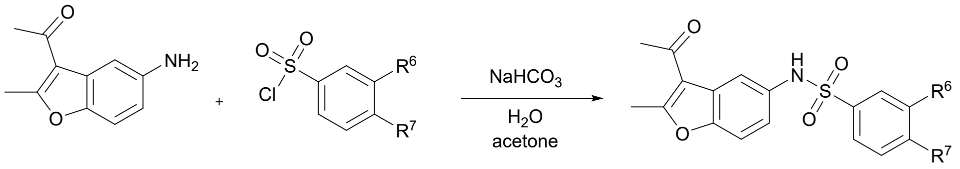

1

B1, R^6 = H, R^7 = CO_2H 50%

B2, R^6 = CO_2H, R^7 = H 63%

B3, R^6 = H, R^7 = $CONH_2$ 34%

B4, R^6 = $CONH_2$, R^7 = H 35%

B5, R^6 = H, R^7 = CO_2Et 66%

B6, R^6 = CO_2Me, R^7 = H 78%

B7, R^6 = Cl, R^7 = H 77%

Figure S1.

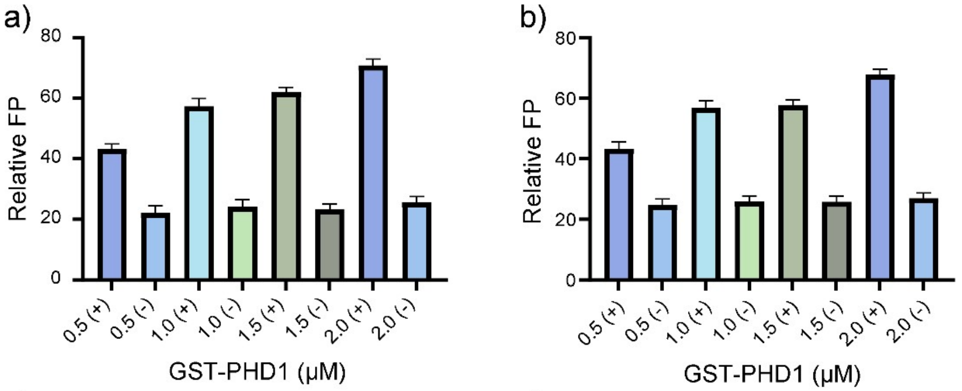

c)

[GST-PHD1] (μM) in buffer	Avg FP ± SD – no BGG	Avg FP ± SD – 0.1% BGG
0.5 (+)	43.3 ± 1.72	43.5 ± 2.04
0.5 (-)	22.2 ± 2.28	24.8 ± 2.07
1.0 (+)	57.4 ± 2.54	57 ± 2.28
1.0 (-)	24.2 ± 2.38	25.9 ± 1.92
1.5 (+)	62.1 ± 1.58	57.8 ± 1.8
1.5 (-)	23.4 ± 1.82	25.8± 1.87
2.0 (+)	71 ± 1.94	67.8 ± 1.82
2.0 (-)	25.8 ± 1.84	27 ± 1.76

d)

[GST-PHD1] (μM)	% BGG	Z′
0.5	-	0.43
0.5	0.1	0.34
1.0	-	0.56
1.0	0.1	0.59
1.5	-	0.74
1.5	0.1	0.66
2.0	-	0.75
2.0	0.1	0.74

Figure S1. Z prime (Z´) assay optimization. Assay optimization included varying the amounts of GST-PHD1 and bovine gamma globulin (BGG) to obtain a robust Z´ before continuing with screening various libraries. a-d) The amount of GST-PHD1 (0.50, 1.0, 1.5 or 2.0 μM) was varied to determine the optimal amount for the best dynamic range in FP signal using the positive control (+) and negative control (-) buffers. In either control, BGG was either omitted (panel a) or added (0.1%, panel b) to ensure its use would not affect the FP signal. a-b) The bar graphs represent the average of each condition (48 samples each) and errors bars represent the SD. c) Summarized results reflected in panels a and b. d) Assay robustness is indicated by the Z´. Summarized resultsdemonstrate that using 1.0 μM GST-PHD1 in buffer with or without BGG maintained a comparable robust Z´ (0.56 vs 0.59).

Figure S2.

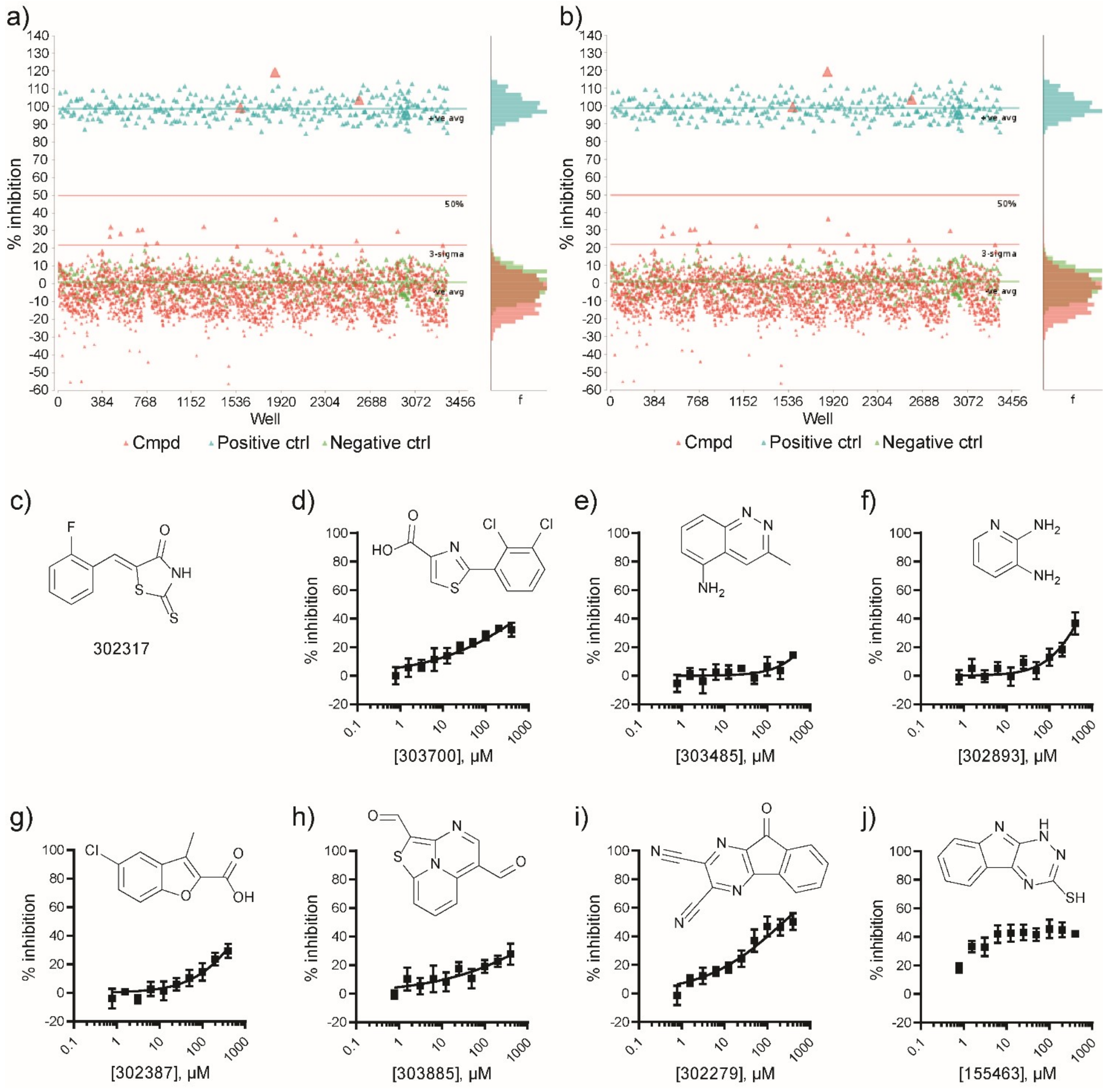

Figure S2. Fluorescence polarization-based fragments screen against GST-PHD1. a,b) Scatter plots of pilot screens with 3,095 fragments tested at 50 µM with 1 µM GST-PHD1, 20 nM 5-FAM H3K4me0 10-mer, 1 mM TCEP, without BGG (a) or with 0.1% BGG as a carrier protein (b). The positive control includes 120 µM unlabeled H3K4me0 10-mer peptide (blue triangles), while DMSO is used as a negative control (green triangles). Red triangles represent the screened compounds and their % inhibition. Green and blue lines indicate mean ± SD of controls. Three SD above the mean inhibition value of the library (~20% inhibition) is represented by the 3-sigma red line. All assays were run in 50 mM HEPES, 50 mM KCl buffer at pH 7.5 with 0.01% Tween-20. c) PAINS compound identified as a hit in the pilot screen with BGG, shown in panel b. d-j) Repurchased fragment hits that displayed dose-dependent inhibition of the interaction between the 5-FAM labeled H3K4me0 peptide and GST-PHD1. Fragments were tested in triplicate at a concentration range of 0.781-400 µM using the HTS assay conditions and are reported as

the mean ± SD. These hits lie between the 3 SD above the mean and the 50% inhibition line on the scatterplot in panel b.

Figure S3.

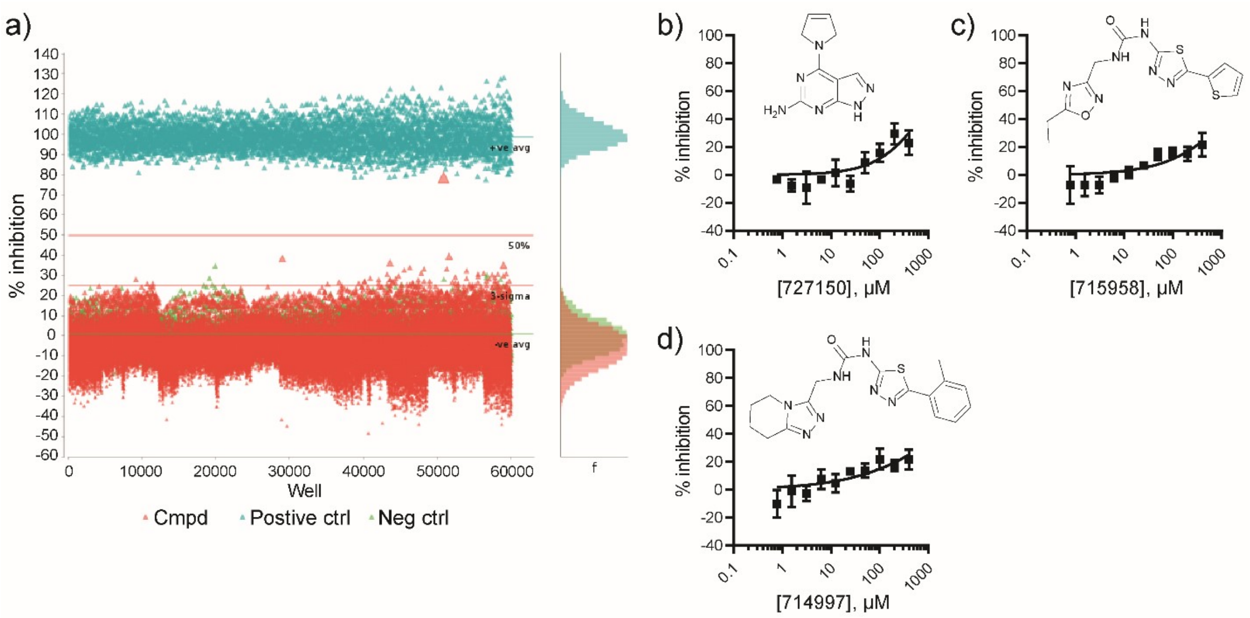

Figure S3. Results of the ChemBridge Premium Diversity library HTS with GST-PHD1. a) Scatter plot of HTS with 50,000 compounds tested at 10 μM with 1 μM GST-PHD1, 20 nM 5-FAM H3K4me0 10-mer peptide, 1 mM TCEP, and 0.1% BGG. DMSO is used as the negative (-) control (green). The positive (+) control includes 120 μM unlabeled H3K4me0 10-mer (blue). Screened compounds and their % inhibition are shown in red. Green and blue lines indicate mean ± SD of controls. Three SD above the mean inhibition value of the library (~20% inhibition) is represented by the 3-sigma red line and determined 66 compounds as hits. FP assays were run in 50 mM HEPES, 50 mM KCl at pH 7.5 with 0.01% Tween-20. b-d) Repurchased ChemBridge screen hits that displayed dose-dependent inhibition of GST-PHD1. Compounds b) 727150, c) 715958, and e) 714997 were tested using a concentration range of 0.781-400 μM using the HTS assay conditions. The data are reported in triplicate from technical replicates as the means ± SD.

Figure S4.

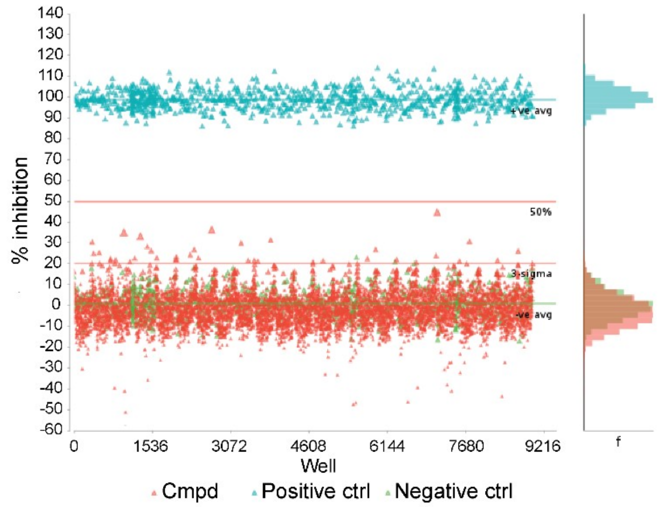

Figure S4. HTS of the NCI collection of libraries including protein-protein interaction inhibitors, macrocycles, and natural products. Scatter plot of HTS with 50,000 compounds tested at 50 µM with 1 µM GST-PHD1, 20 nM 5-FAM H3K4me0 10-mer peptide, 1 mM TCEP, and 0.1% BGG. DMSO is used as the negative (-) control (green). The positive (+) control includes 120 µM unlabeled H3K4me0 10-mer (blue). Screened compounds and their % inhibition are shown in red. Green and blue lines indicate mean ± SD of controls. Three SD above the mean inhibition value of the library (~20% inhibition) is represented by the 3-sigma red line. FP assays were run in 50 mM HEPES, 50 mM KCl at pH 7.5 with 0.01% Tween-20. Thirty-eight compounds displayed % inhibition 3 SD above the mean.

Figure S5.

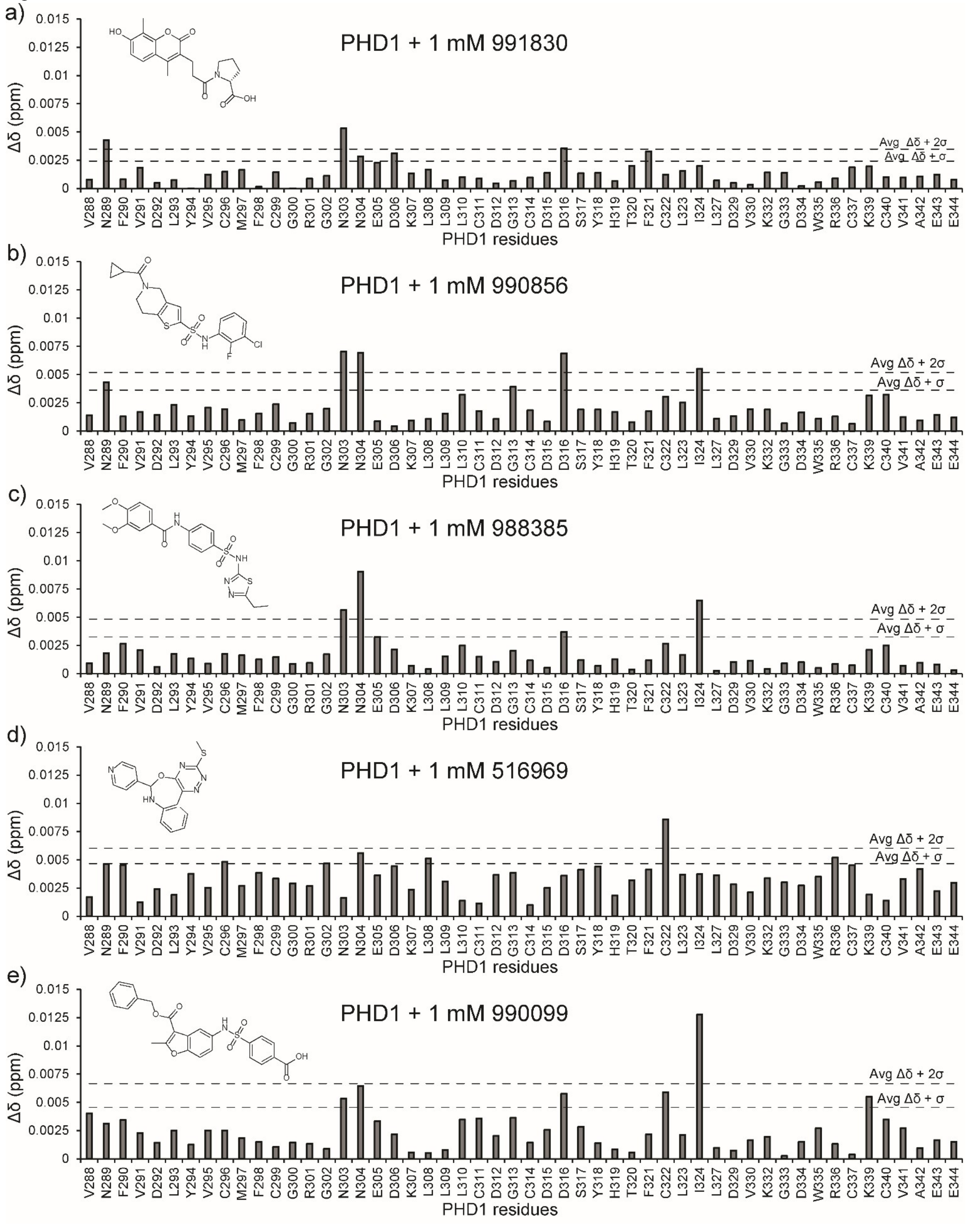

a)
PHD1 + 1 mM 991830
b)
PHD1 + 1 mM 990856
c)
PHD1 + 1 mM 988385
d)
PHD1 + 1 mM 516969
e)
PHD1 + 1 mM 990099
Δδ (ppm)
PHD1 residues
Avg Δδ + 2σ
Avg Δδ + σ

Figure S5. ^{1}H-^{15}N PHD1 (50 µM) chemical shift perturbations (CSP) induced by 1 mM of NCI screen hits. PHD1 residues affected by 1 mM of compound a) 991830, b) 990856, c) 988385, d) 516969, and e) 990099 are shown. Dashed lines represent the average CSP plus one standard deviation (Avg Δδ + σ) or the average CSP plus two standard deviations (Avg Δδ + 2σ).

Figure S6.

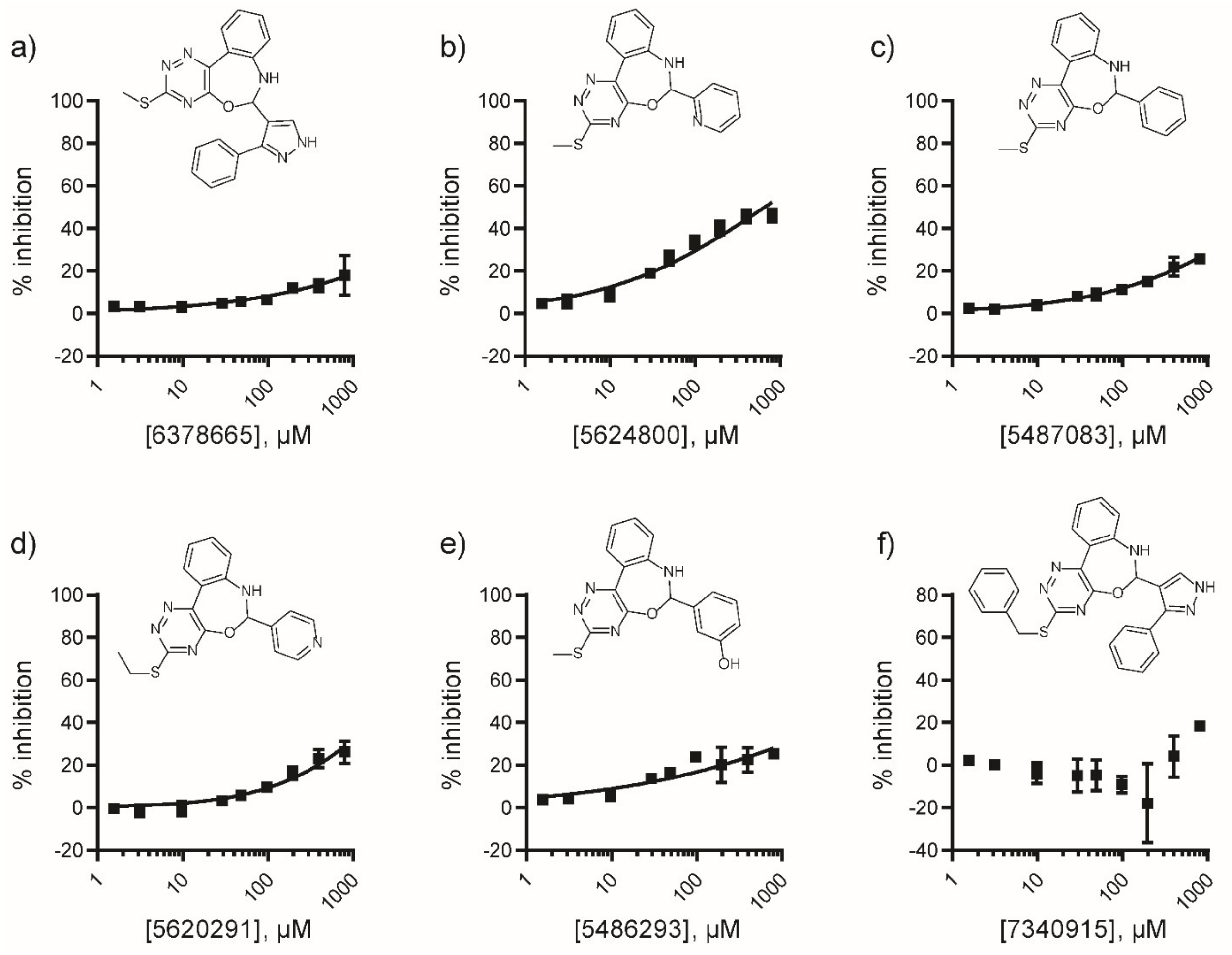

Figure S6. FP concentration-response curves of derivatives of NCI screen hit 516969. Compounds a) 6378665, b) 5624800, c) 5487083, d) 5620291, e) 5486293, and f) 7340915 were tested using a concentration range of 1.55-800 µM using the HTS assay conditions. Data were collected in quadruplicate and display the mean ± SD.

Figure S7.

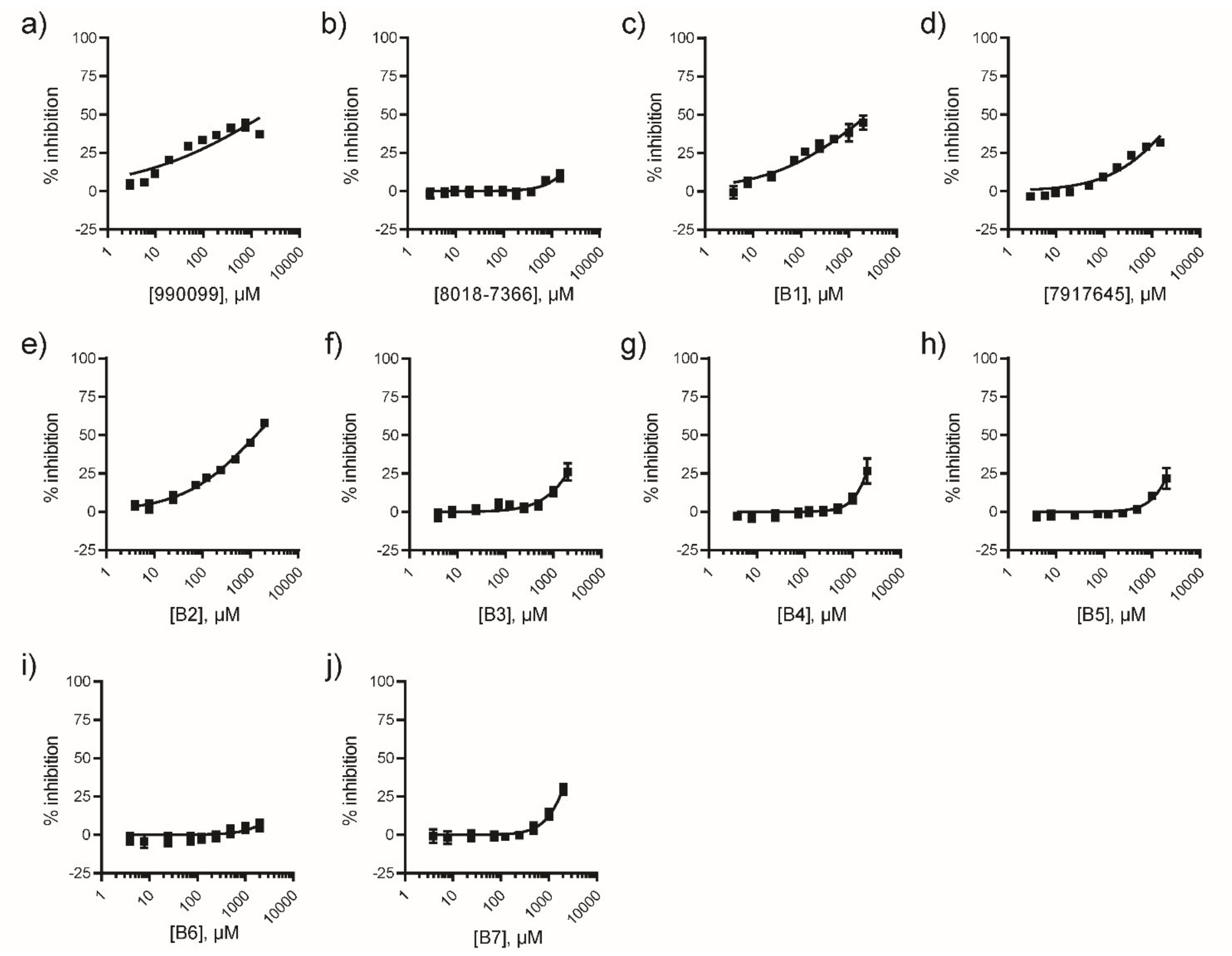

Figure S7. FP dose-dependent inhibition of GST-PHD1 by select analog-by-catalog derivatives of SMDC 990099. a-b) Compounds a) 990099 and b) 8018-7366 were evaluated using a concentration range of 2.9-1500 µM. c-j) Compounds c) B1, d) 7917645, e) B2, f) B3, g) B4, h) B5, i) B6, j) B7 were tested using a concentration range of 3.9-200 µM compound. Data were collected in quadruplicate and display the mean ± SD (n = 1 for compounds 990099 and 8018-7366; n = 2 for compounds 7917645 and B1-B7).

Figure S8.

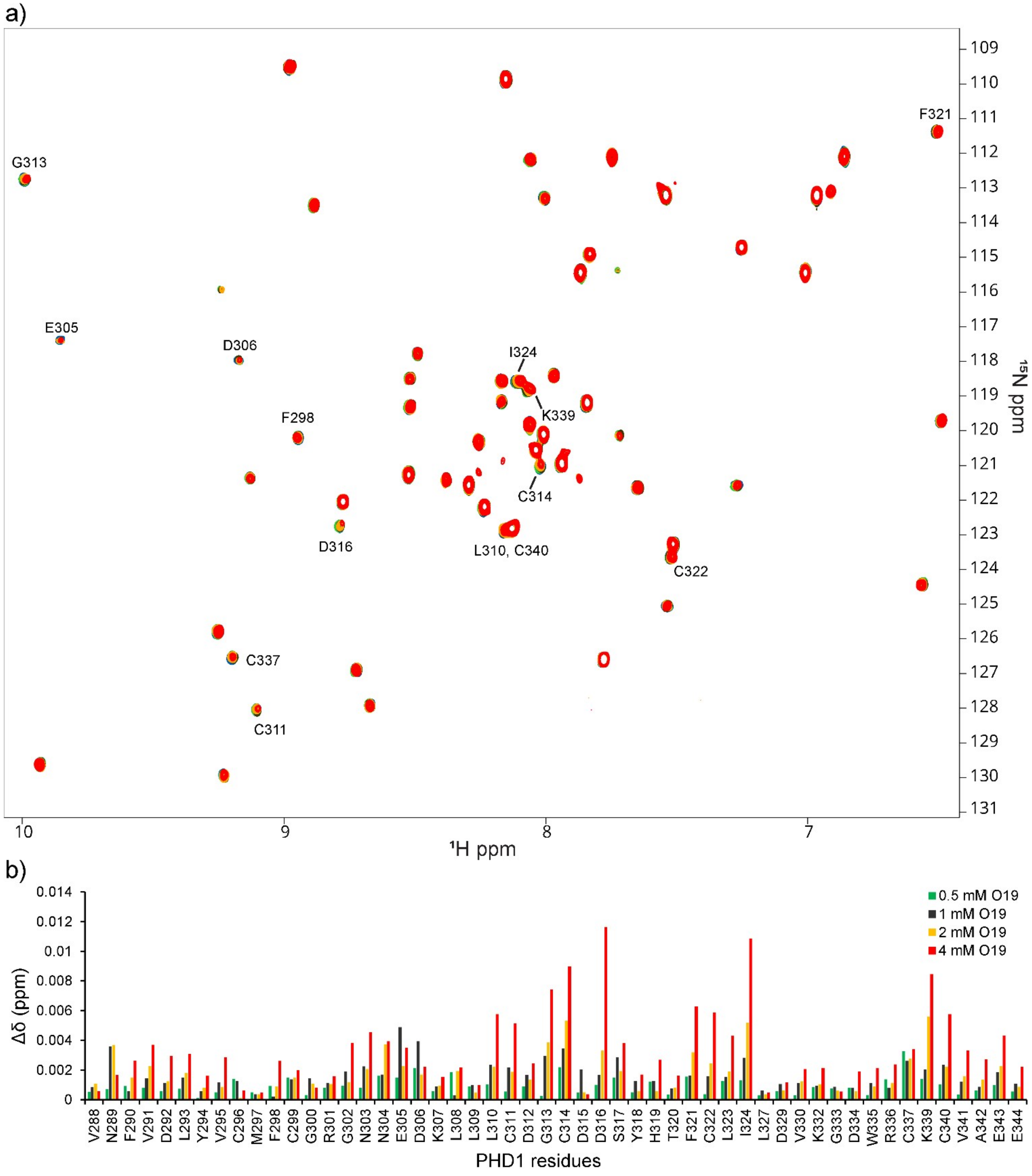

Figure S8. ${}^{1}H$-${}^{15}N$ HSQC NMR spectra displaying titration of B2 with 50 µM ${}^{15}N$-labeled PHD1. a) Overlay of HSQC spectra of apo PHD1 with DMSO (blue) compared to PHD1 with 0.5 mM (green), 1 mM (black), 2 mM (orange), and 4 mM (red) B2. b) CSPs values of PHD1 resonances perturbed by increasing concentration of B2.

Supporting Spectra

Spectrum S1. ^{1}H NMR of B1

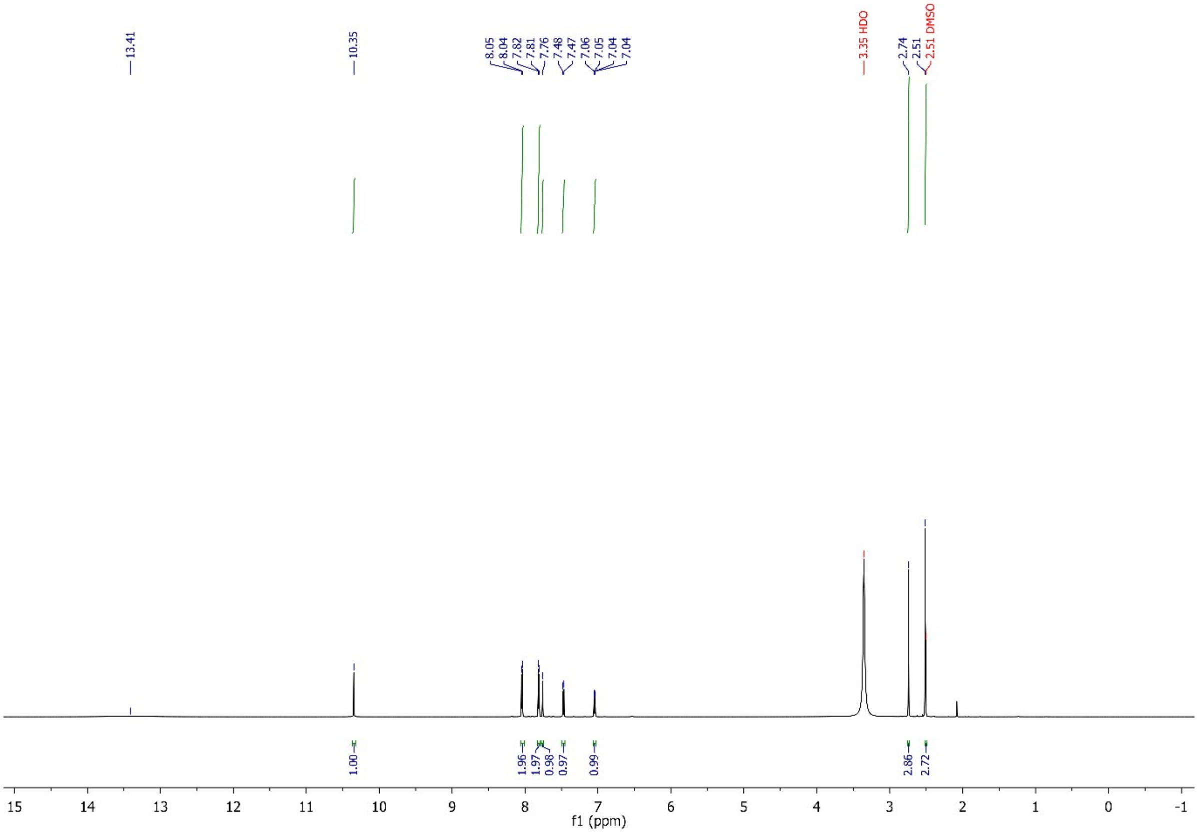

Spectrum S2. ^{13}C NMR of B1

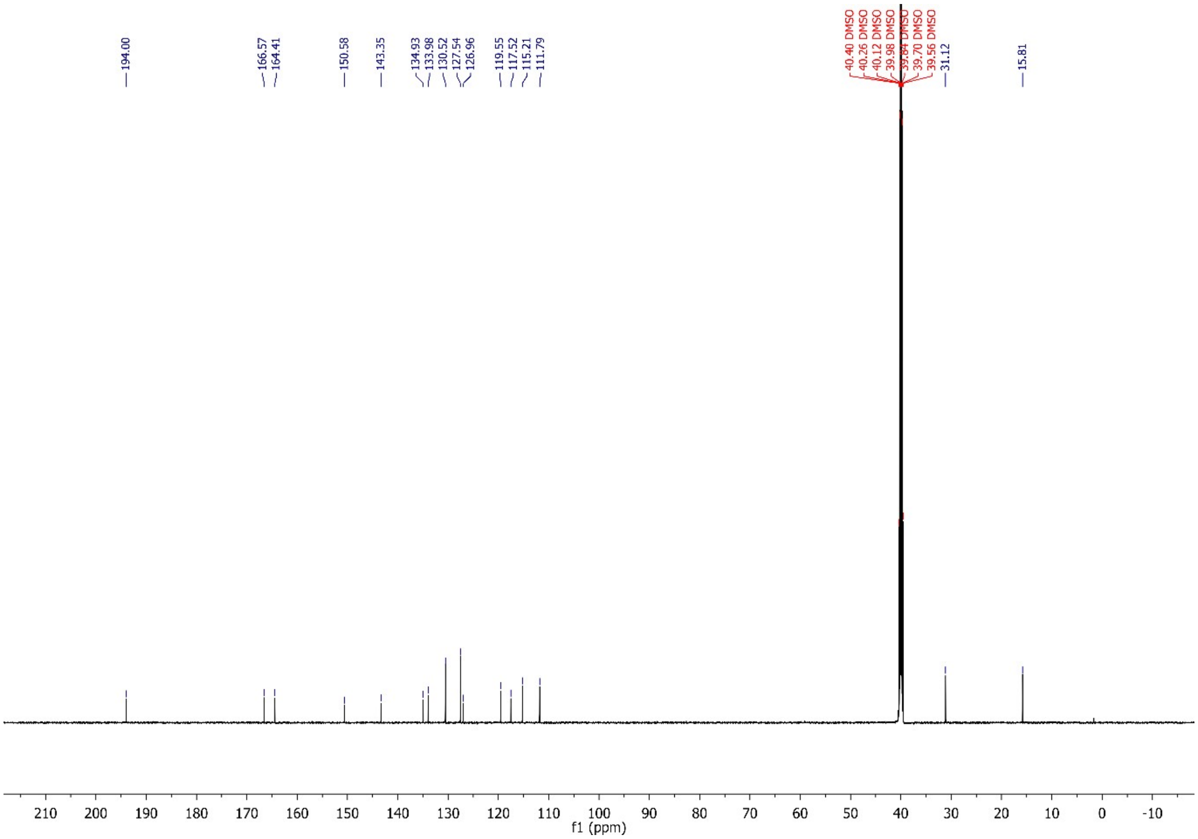

Spectrum S3. ^{1}H NMR of B2

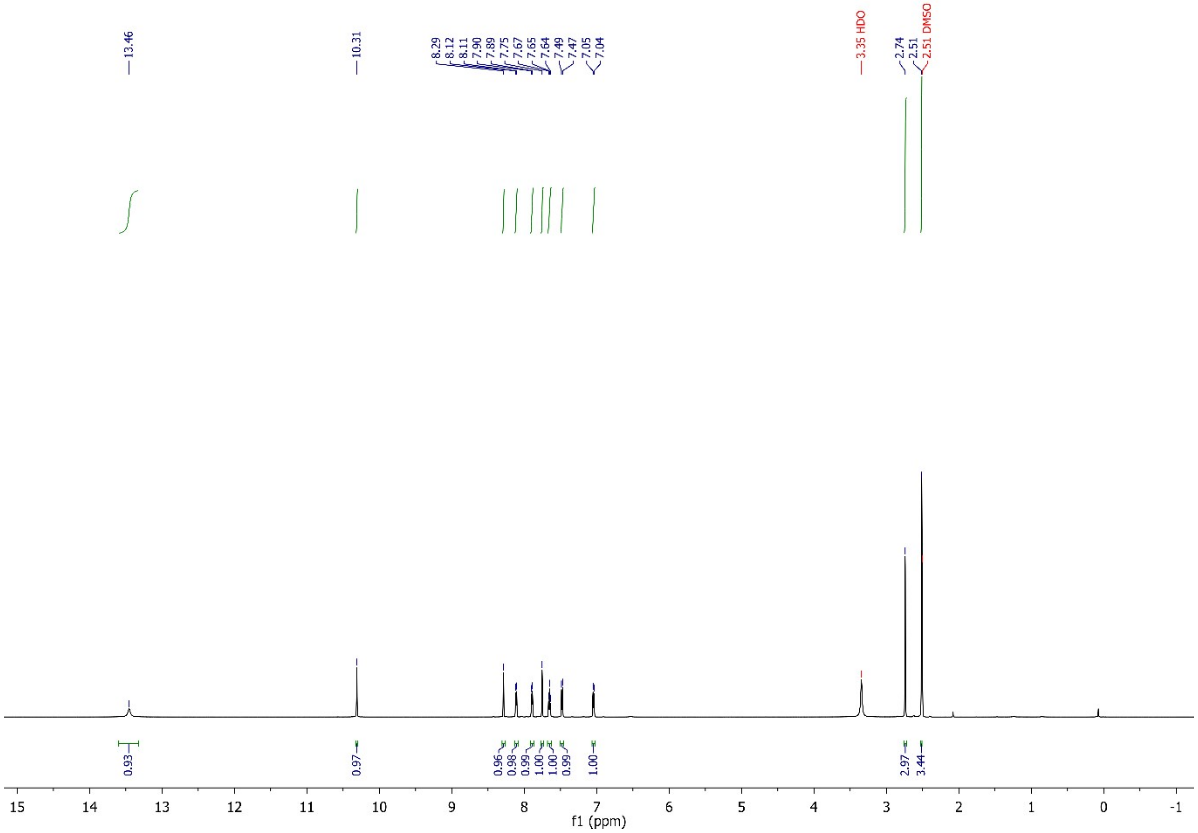

Spectrum S4. ^{13}C NMR of B2

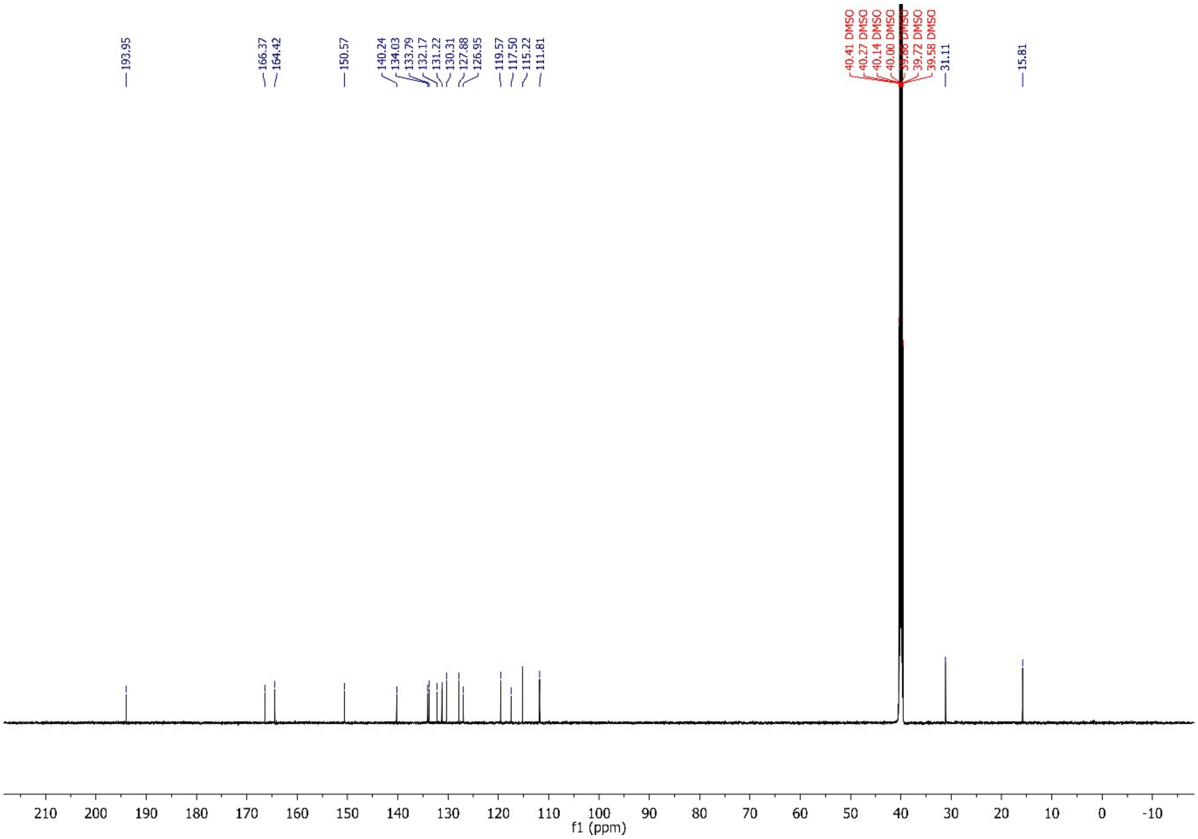

Spectrum S5. ^{1}H NMR of B3

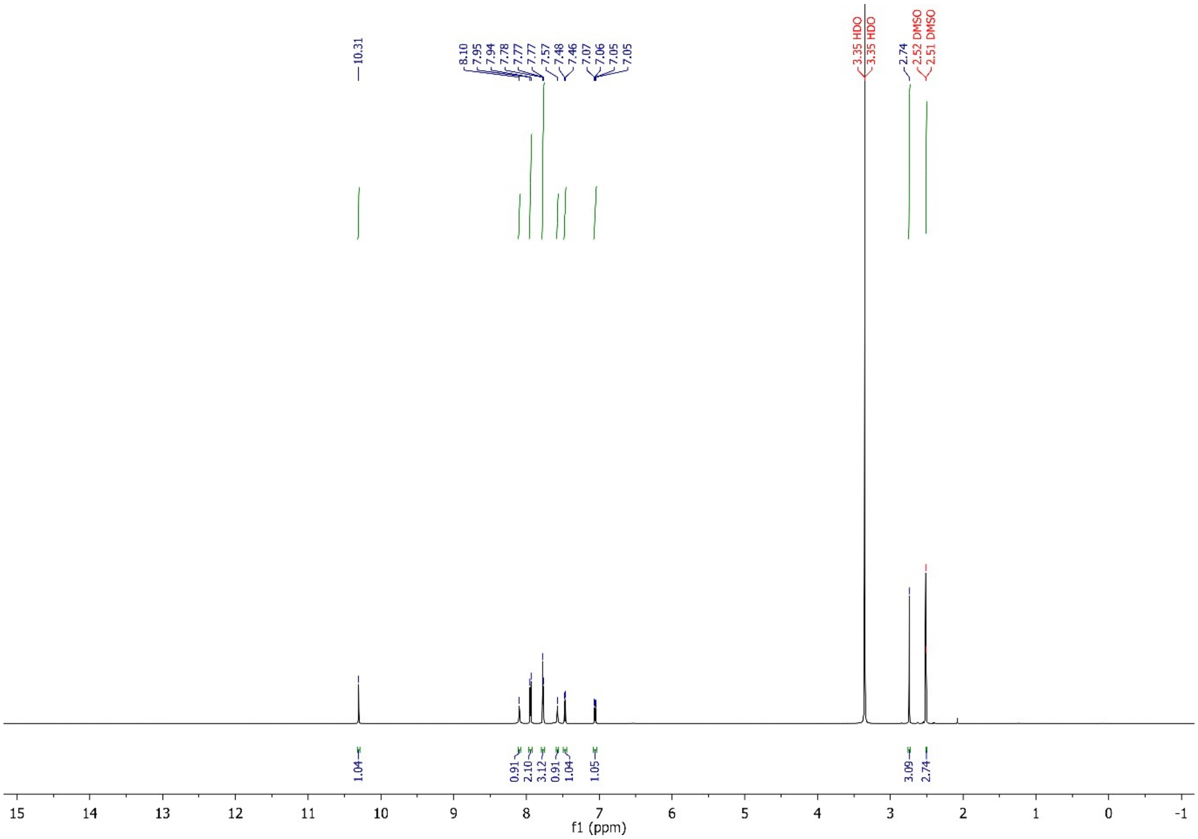

Spectrum S6. ^{13}C NMR of B3

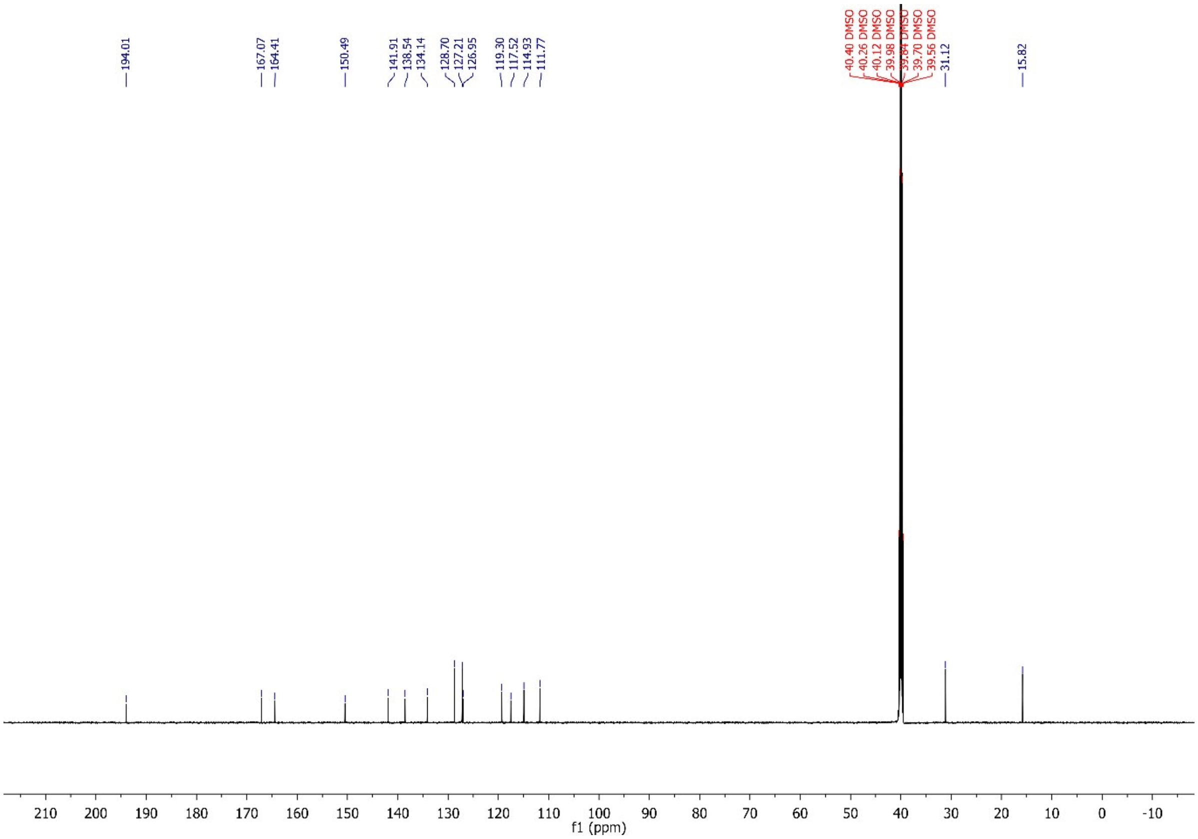

Spectrum S7. ^{1}H NMR of B4

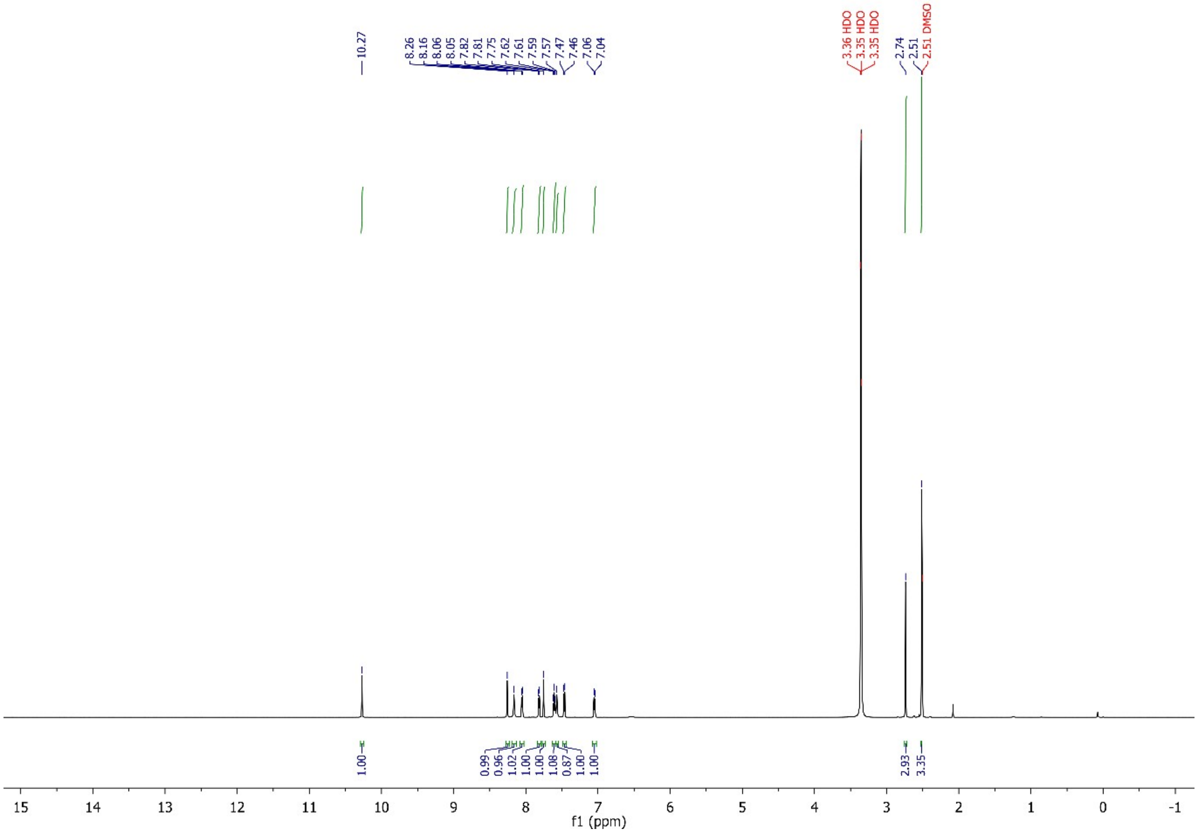

Spectrum S8. ^{13}C NMR of B4

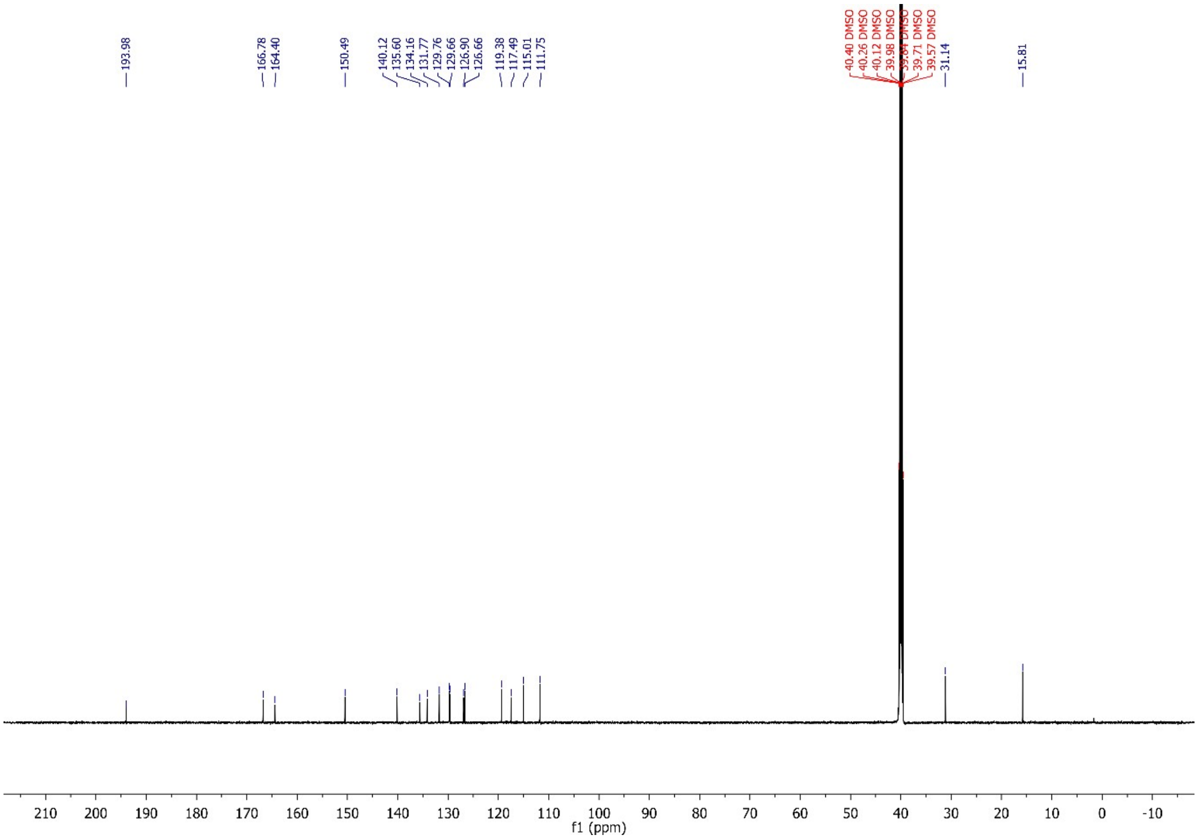

Spectrum S9. ^{1}H NMR of B5

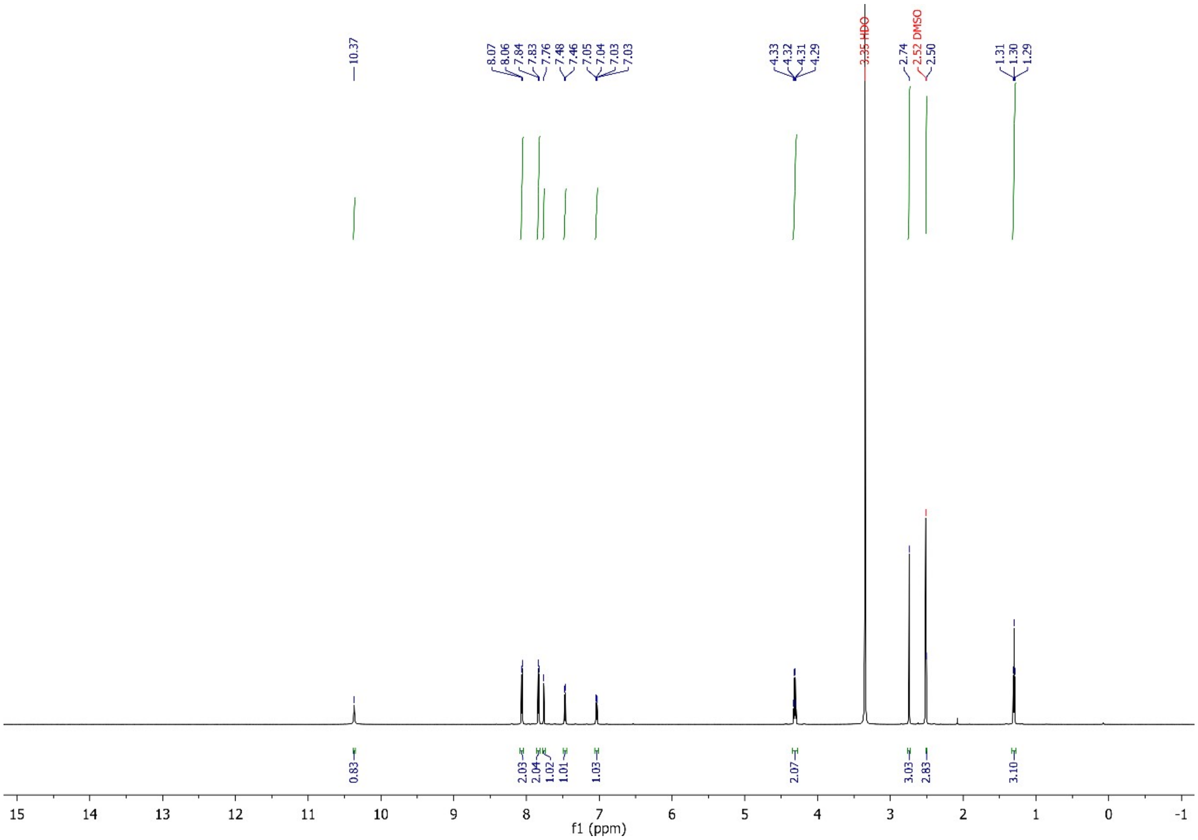

Spectrum S10. ^{13}C NMR of B5

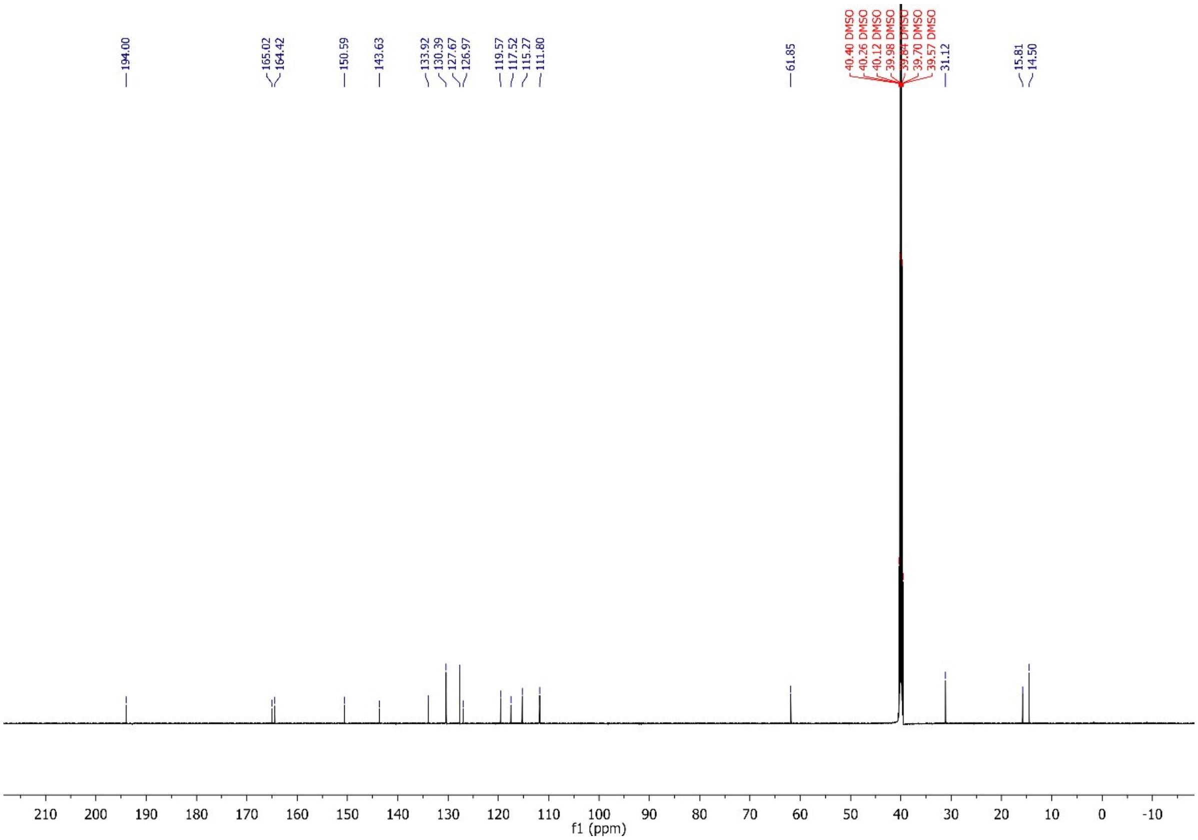

Spectrum S11. ^{1}H NMR of B6

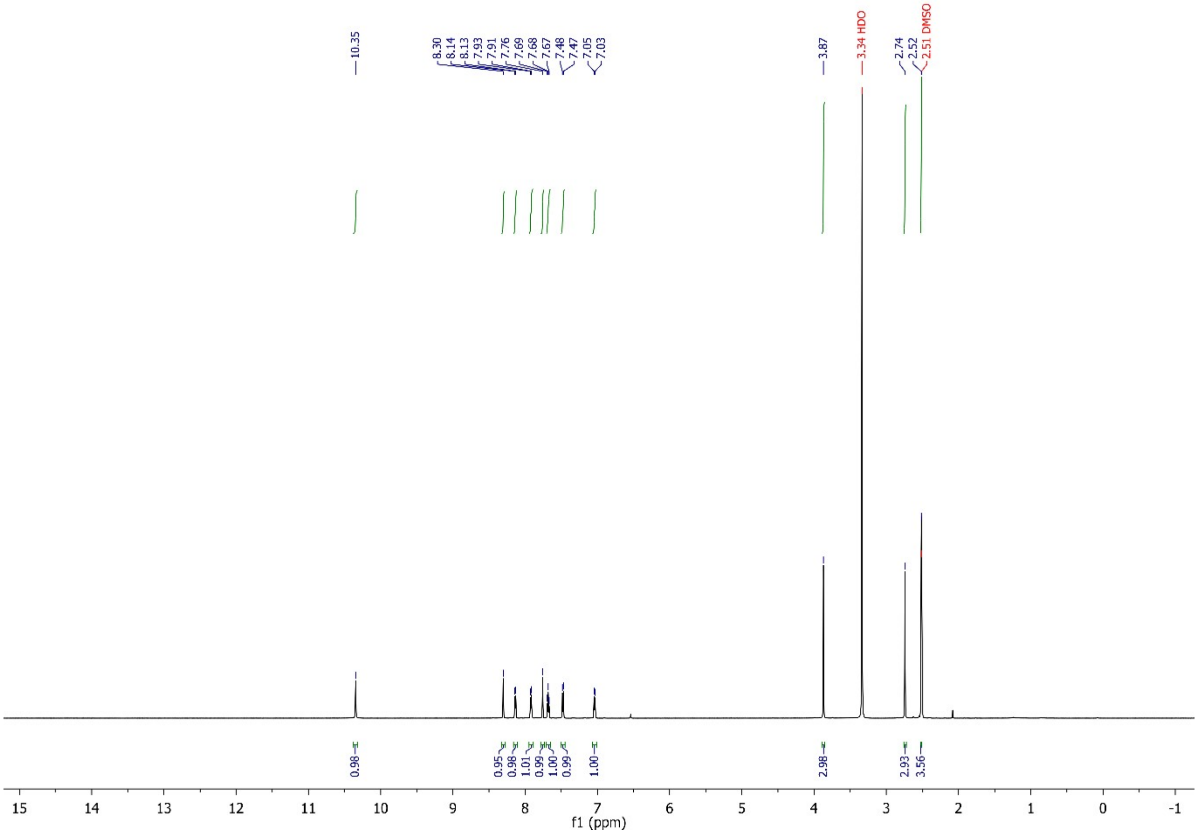

Spectrum S12. ^{13}C NMR of B6

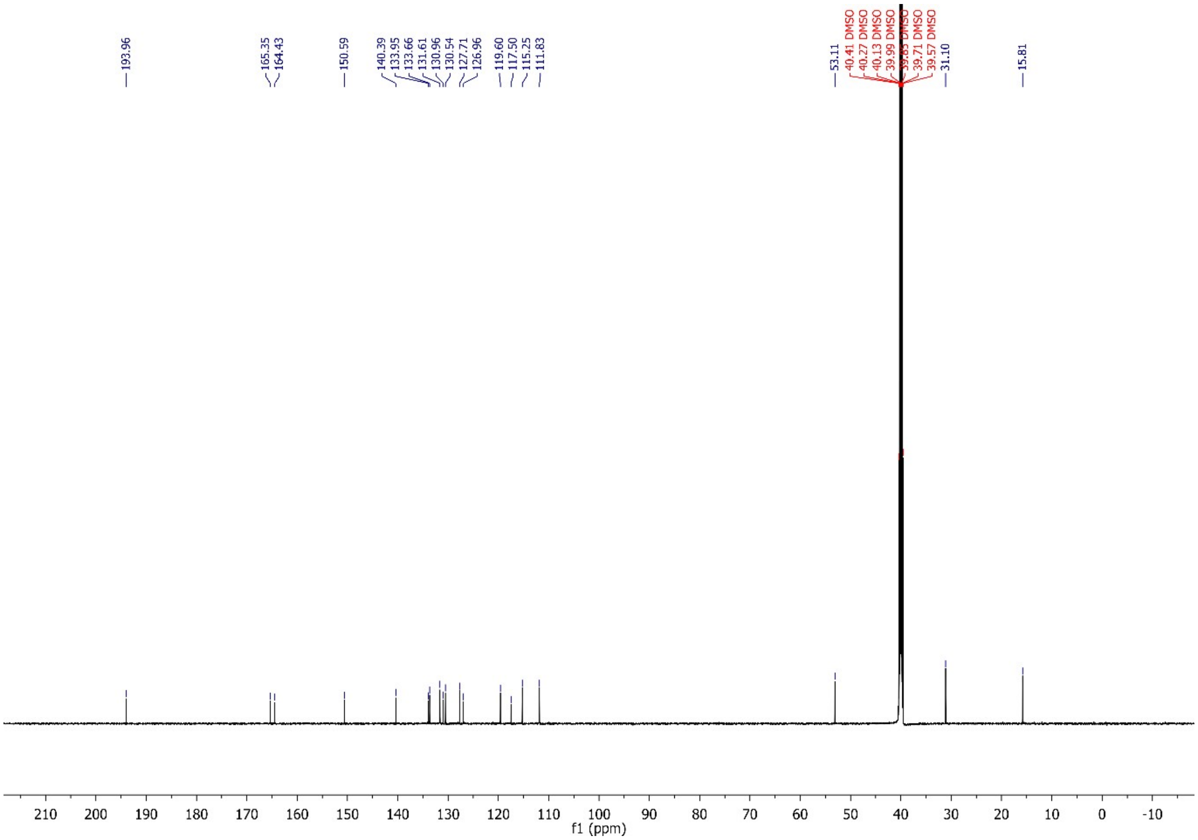

Spectrum S13. ^{1}H NMR of B7

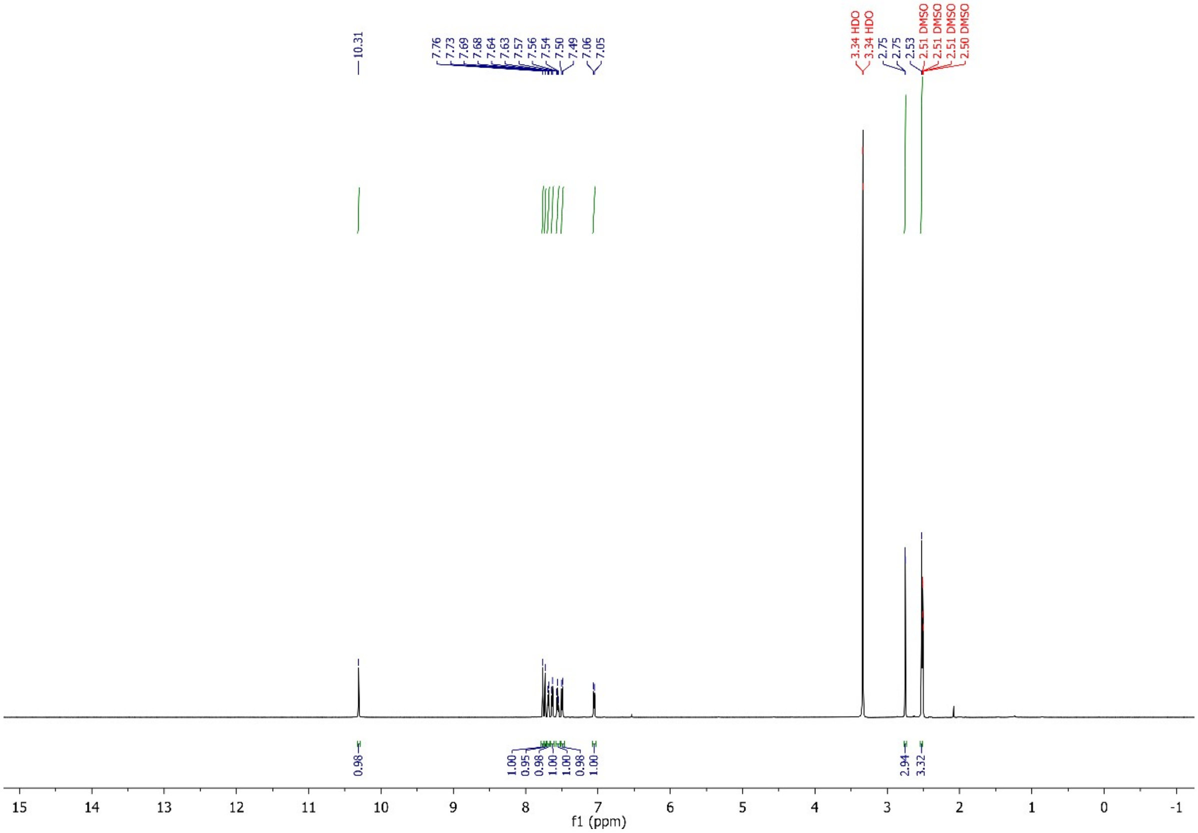

Spectrum S14. ^{13}C NMR of B7

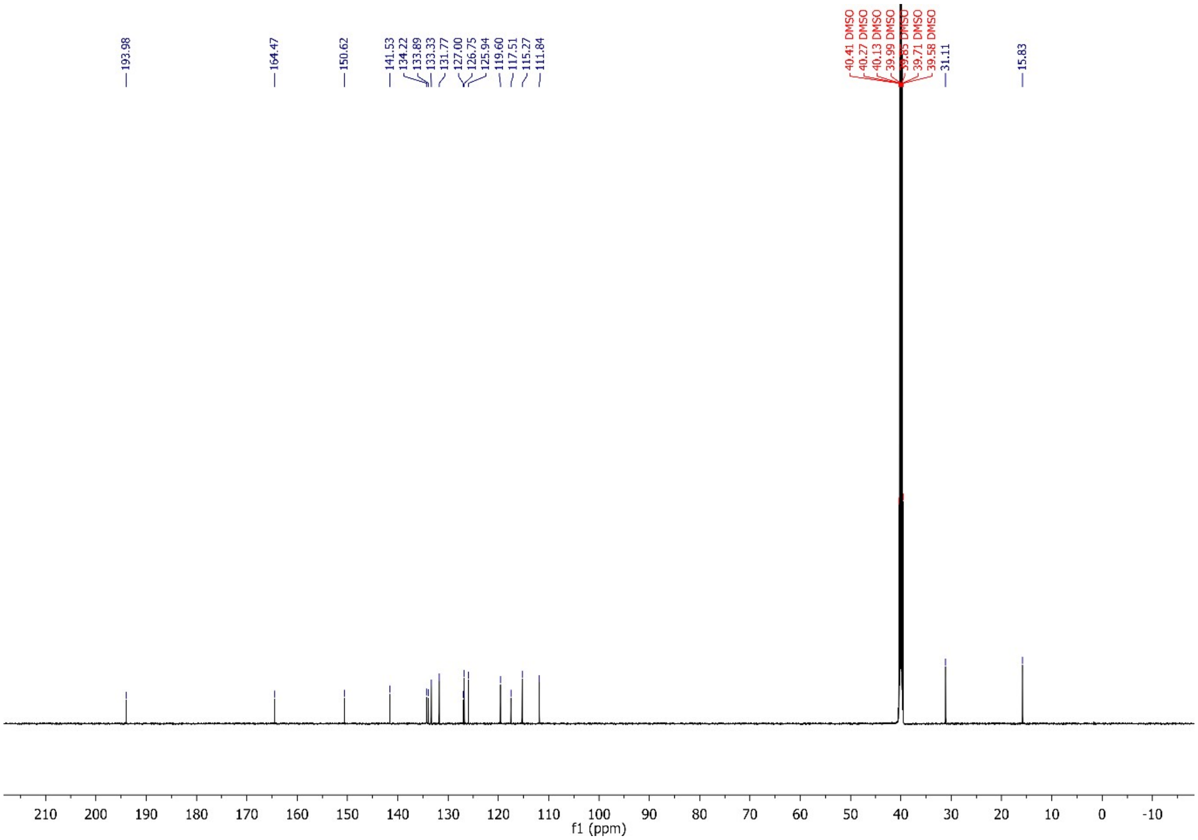

Supplementary references

1 V. M. Lyubchanskaya, E. K. Panisheva, S. A. Savina, L. M. Alekseeva, A. S. Shashkov and V. G. Granik, *Russ. Chem. Bull.*, 2005, **54**, 1690–1699.

2 S. A. Patil, P. A. Medina, D. Gonzalez-Flores, J. K. Vohs, S. Dever, L. W. Pineda, M. L. Montero and B. D. Fahlman, *Synth. Commun.*, 2013, **43**, 2349–2364.

3 J. E. Longbotham, C. M. Chio, V. Dharmarajan, M. J. Trnka, I. O. Torres, D. Goswami, K. Ruiz, A. L. Burlingame, P. R. Griffin and D. G. Fujimori, *Nat. Commun.*, 2019, **10**, 1–12.

4 J. E. Longbotham, M. J. S. Kelly and D. G. Fujimori, *ACS Chem. Biol.*, 2023, **18**, 1915–1925.

5 J.-H. Zhang, T. D. Y. Chung and K. R. Oldenburg, *J. Biomol. NMR*, 1999, **4**, 67–73.

6 T. Sander, J. Freyss, M. Von Korff and C. Rufener, *J. Chem. Inf. Model.*, 2015, **55**, 460–473.

7 L. Mureddu and G. W. Vuister, *FEBS J.*, 2019, **286**, 2035–2042.
